# Single‐cell transcriptomics provides insights into the origin and microenvironment of human oesophageal high‐grade intraepithelial neoplasia

**DOI:** 10.1002/ctm2.874

**Published:** 2022-05-24

**Authors:** Guobin Liao, Nan Dai, Tiantian Xiong, Liang Wang, Xinwei Diao, Zhizhen Xu, Yuanli Ni, Dingrong Chen, Airui Jiang, Hui Lin, Shuangshuang Dai, Jianying Bai

**Affiliations:** ^1^ Department of Gastroenterology the Second Affiliated Hospital Army Medical University Chongqing China; ^2^ Cancer Center Daping Hospital Army Medical University Chongqing China; ^3^ Department of Biochemistry and Molecular Biology Army Medical University Chongqing China; ^4^ Pathology the Second Affiliated Hospital Army Medical University Chongqing China; ^5^ Chongqing University Cancer Hospital Chongqing China

**Keywords:** heterogeneity, high‐grade intraepithelial neoplasia, single‐cell RNA sequencing, tumour origin

## Abstract

**Background:**

High‐grade intraepithelial neoplasia (HIN) is the precursor of oesophageal squamous cell carcinoma. The molecular and functional properties of HIN are determined by intrinsic origin cells and the extrinsic microenvironment. Yet, these factors are poorly understood.

**Methods:**

We performed single‐cell RNA sequencing of cells from HINs and adjacent tissues from the human oesophagus. We analysed the heterogeneity of basal layer cells and confirmed it using immunostaining. Aneuploid cells in HIN were studied using primary cell culture combined with karyotype analysis. We reconstructed the lineage relationship between tumour and normal populations based on transcriptome similarity. Integration analysis was applied to our epithelial data and published invasive cancer data, and results were confirmed by immunostaining and 3D organoid functional experiments. We also analysed the tumour microenvironment of HIN.

**Results:**

The basal layer contained two cell populations: KRT15^high^STMN1^low^ and KRT15^high^STMN1^high^ cells, which were located mainly in the interpapillary and papillary zones, respectively. The KRT15^high^STMN1^low^ population more closely resembled stem cells and transcriptome similarity revealed that HIN probably originated from these slow‐cycling KRT15^high^STMN1^low^ cells. 3D Organoid experiments and RNA‐sequencing showed that basal‐cell features and the differentiation ability of the normal epithelium were largely retained in HIN, but may change dramatically in tumour invasion stage. Moreover, the tumour microenvironment of HIN was characterised by both inflammation and immunosuppression.

**Conclusions:**

Our study provides a comprehensive single‐cell transcriptome landscape of human oesophageal HIN. Our findings on the origin cells and unique microenvironment of HIN will allow for the development of strategies to block tumour progression and even prevent cancer initiation.

## Graphical abstract

• A comprehensive single‐cell transcriptome landscape of human oesophageal high‐grade neoplasia.

• Basal layer cells are heterogeneous by transcriptome and location: KRT15^high^STMN1^low^ and KRT15^high^STMN1^high^ cells, which are located mainly at interpapillary zone and papillary zone, respectively.

• HIN probably originated from slow‐cycling KRT15^high^STMN1^low^ cells.

• HIN is slightly biased in the transcriptome, which may change dramatically in tumour invasion stage.

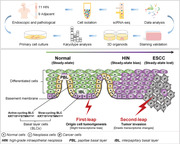

**Abbreviations used in this paper**
ABC: active‐cycling basal cell
BLC: basal layer cell
DEG: differentially expressed gene
ESCC: oesophageal squamous cell carcinoma
HIN: high‐grade intraepithelial neoplasia
IBL: interpapillary basal layer
PBL: papillae basal layer
SBC: slow‐cycling basal cell
scRNA‐seq: single‐cell RNA sequencing

## 1 INTRODUCTION

High‐grade intraepithelial neoplasia (HIN) is a precursor to oesophageal squamous cell carcinoma (ESCC). It harbours mutations and genomic instability similar to ESCC^1^ and is associated with a very high risk of ESCC. In patients with HIN, the cumulative incidence of ESCC is up to 73.9–75.0% during a median follow‐up of 13.5 years.^2^ Over the past decade, a large number of HINs have been detected and treated using cutting‐edge endoscopic techniques, which is recognised in an effort to reduce ESCC mortality.^3^ As a consequence, the demand for early precision diagnosis and personalised treatments has increased exponentially. This demand emphasises the urgent need to investigate the cellular and molecular characteristics of the initiation and early development of HIN.

Bulk resolution sequencing and missing single‐cell profiles reveal that human HIN and ESCC carry similar mutations and copy number variations (CNVs). Mutations in TP53 and CNV gains on chromosome 3q may be early events in such carcinomas.^1^, ^4^ These studies provide valuable insights into oesophageal HIN. Additionally, research has suggested that mouse oesophageal HIN may originate from basal layer cells (BLCs).^5^, ^6^, ^7^, ^8^ Yet, there is significant heterogeneity between BLCs in mouse^9^, ^10^, ^11^, ^12^, ^13^ and human esophagus,^11^, ^14^, ^15^, ^16^, ^17^ and it would be presumptuous to suggest that human and rodent neoplasias behave similarly. In a carcinogen‐induced murine model, an immune response transition from TH1 to TH17 may promote the early progression of HIN.^18^ Yet, this model cannot be entirely consistent with the chronic nature of human HIN, which has not been deliberately induced. Hence, due to the substantial species differences between rodents and humans (e.g. the mouse oesophagus lacks papillae), the cellular and molecular features of human oesophageal HIN are not fully understood. This emphasises the importance of high‐resolution research on human oesophageal HIN tissues.

To address these issues, we applied single‐cell RNA sequencing (scRNA‐seq) to cells from HIN tissues and adjacent normal tissues of the human oesophagus and generated a comprehensive transcriptome atlas. Our data provided novel and solid evidence for the heterogeneity of oesophageal BLCs, by transcriptome and location. By analysing and comparing the transcriptome profiles of HIN and normal squamous epithelial cells, we determined the specific cellular origin of human HIN. Moreover, we found the multistep changes in the transcriptome of tumour early stage, which are important for understanding HIN initiation and early progression. We further depicted the microenvironment ecosystem of non‐epithelial cells, which was different from that of mouse HIN and human ESCC. Our study provides orthogonal lines of evidence for early precision diagnosis and prevention strategies for human oesophageal HIN at the cellular and molecular level, which will also have important implications in the prevention of ESCC.

## 2 METHODS

### 2.1 Ethics statement and human biospecimen collection

Human oesophageal tissues were obtained from Xinqiao Hospital (Chongqing, China), with written informed consent. The study was approved by the Medical Ethics Committee of Xinqiao Hospital of the Army Medical University, PLA (NO.2019‐100‐01), in accordance with the Declaration of Helsinki. Immediately after the samples were obtained (ten HINs from endoscopic mucosal dissection, one HIN, and nine adjacent tissues from biopsy), part of the fresh HIN tissues were dissected for enzymatic digestion into single cells as described below, and the remaining tissues were fixed with 4% paraformaldehyde solution and embedded in paraffin. The adjacent normal tissues were measured to be approximately 3 cm from the tumour edge using Narrow Band Imaging combined with Magnifying Chromoendoscopy. Separately paired HIN and invasive cancer samples were used for primary cell culture (including organoid culture) and subsequent karyotype analysis. The histological results were independently validated by two experienced pathologists.

### 2.2 Preparation of single‐cell suspensions

HIN tissues for scRNA‐seq were washed with phosphate‐buffered saline (PBS; Thermo Fisher Scientific), cut into small pieces (<1 mm^3^) on ice and digested using a Tumor Dissociation Kit (Miltenyi Biotec, 130‐095‐929), according to the manufacturer's instructions. After filtration, the cell pellet was collected by centrifugation at 500 g for 5 min at 4°C. The cell pellet was then washed three times in wash buffer (0.32 M of sucrose; Sigma‐Aldrich, and 5 mM of CaCl_2_, Sigma‐Aldrich, 10043‐52‐4) and resuspended in a storage buffer (0.43 M of sucrose; Sigma‐Aldrich, and 70 mM of KCl; Ambion, AM9640G). Next, the cells were counted and assessed for viability using trypan blue staining with a haemocytometer.

### 2.3 scRNA‐seq library preparation and pre‐processing

scRNA‐seq was performed using the 10× Genomics Chromium Single‐Cell 3′ Kit (v.2) according to the manufacturer's instructions. The sequencing reads were mapped to the hg38 human reference genome built using Cell Ranger v.3.1.0 (10× Genomics), and count tables of unique molecular identifiers (UMIs) were generated for each gene per cell. We performed principal component analysis (PCA) and uniform manifold approximation and projection (UMAP) dimensionality reduction with Seurat. The Seurat functions ‘FindNeighbors’ and ‘FindClusters’ were used to identify clusters of cells with similar transcriptomes based on their PCs (resolution = 0.5).

### 2.4 Quality control and normalisation of single‐cell data

The Seurat R package (v.3.0.2) was used to calculate quality control metrics.^19^ Cells with maximum value UMIs greater than 90%, and less than 200 genes per cell were removed. Cells with a mitochondrial expression percentage greater than 25% were also removed. We normalised using the NormalizeData function from Seurat with parameters: ‘scale.factor = 1000; normalization.method = LogNormalize’. We removed the batch effect using the canonical correlation cnalysis (CCA) and mutual nearest neighbours (MNNs) approaches and integrated the batches.

### 2.5 Identification of the major populations and their subpopulations

Following normalisation, 2000 highly variable genes were used as input for PCA. The first 30 principal components were estimated using elbow plots. These principal components were then used to calculate UMAP embeddings and cell clusters were identified based on their PCs, resolution = 0.5. We annotated the major populations using marker genes in conjunction with canonical cell type markers.^20^, ^21^, ^22^ Especially, different highly proliferating cell types (MKI67 and TOP2A) were clustered together for their proliferative activity. After reclustering the main populations with the same technique described above, we manually defined the subpopulations according to marker genes and cell type markers from the literature. Subpopulations with unexplained differentially expressed genes were defined as ‘unknown’ and removed from further analysis. Their marker genes are not canonical cell‐type markers of major populations. For example, cells with high expression of DCN and COL1A2 genes, which are often present in fibroblasts, appeared in the myeloid subpopulations. The number of cells in these subpopulations was low and there was no significant difference between tumour and normal cells in these subpopulations.

### 2.6 Differentiation potential evaluation and transition trajectories analysis

R package cytoTRACE (v.0.3.3)^23^ was used to assess the differentiation potential of cells using scRNA‐seq data and cell populations with high differentiation potential were selected as the starting point for the trajectory.^24^ Diffusion map embeddings were calculated using the destiny R package^25^ (v.3.4.1). Slingshot R package^26^ (v.1.8.0) was used to construct single‐cell transition trajectories based on cell populations identified by Seurat. The monocle2 R package^27^ (v.2.18.0) was used to verify the trajectories.

### 2.7 Transcription factor activity analysis in single cells

Transcription factor (TF) activity analysis was conducted as described by Aibar et al.^28^ We used the pySCENIC (version 0.11.2), which including RcisTarget, GRNboost and AUCell function, to search against the hg38_refseq‐r80_500 bp_up_and_100 bp_down_tss databases (https://resources.aertslab.org/cistarget/) for predicting TF activity. The input matrix was the normalised expression matrix from Seurat.

### 2.8 Discrimination between malignant and non‐malignant cells

We used the R package copyKAT (v.1.0.4) to identify malignant cells as described by Gao et al.^29^ Briefly, copyKAT combines a Bayesian approach with hierarchical clustering to calculate aneuploid copy number events from single cells, which is a unique property of solid tumour cancer cells. We also used the R package inferCNV^30^ (V1.6.0) to identify copy number alterations, which were referenced to fibroblasts. A few cells from normal cells were identified as malignant by inferCNV.

### 2.9 Gene set variation analysis (GSVA)

Gene sets were downloaded from the Molecular Signature Database (MSigDB)^31^ and Cancer Single‐cell State Atlas (CancerSEA).^32^ The pathway scores of each cell were calculated using the GSVA function in the GSVA package^33^ (version 1.38.2) and differential pathway analysis was assessed by the limma R software package^34^ (version 3.46.0).

### 2.10 Activities of metabolic pathways

Metabolism‐related gene sets (from KEGG) were downloaded from the Molecular Signature Database (MSigDB).^31^ Since metabolite concentration and metabolic flow information were not detected in scRNA‐seq data, we referred to Tao Zhang's^35^ method to indirectly evaluate the metabolic activity of single cells. For this, we evaluated the mean expression level of the metabolic gene to characterise the metabolic activity of individual cells.

### 2.11 Hierarchical clustering

To study hierarchical relationships among cell clusters, the Euclidean distance was calculated based on the average expression of each cluster. The ward.D2 clustering method within the hclust function was used to build a dendrogram of the clusters.

### 2.12 Tissue preference analysis

To quantify the tissue preference of each cell cluster, we used the cell number ratio (observed/expected) to measure the enrichment of cells as described by Zhang et al.^36^ Given the sample's contingency table by clusters, we first applied a chi‐square test to assess whether the cell distribution of a sample across clusters or subclusters deviates significantly from random expectations. Then, we calculated the R_o/e_ values for each sample and cluster/subcluster combination as follows: R_o/e _= observed/expected (where ‘observed’ and ‘expected’ represent the observed and expected cell number of a given cluster and sample combination, respectively). The R_o/e_ indicates whether a certain sample of cells is more abundant in a particular cluster or subcluster, and removes the technical variations in estimating tissue preference.^36^, ^37^, ^38^

### 2.13 Cell–cell communication analysis

The python package CellPhoneDB (v.2.1.7) was used to analyse potential interactions across different cell clusters.^39^ Enriched ligand–receptor interactions between any two cell clusters were determined by evaluating the normalised expression levels of annotated ligands and receptors. We defined relevant ligand–receptor interactions only for interactions with values ≥1 with *p*‐values ≤ 0.05. Specifically, we used the difference value of interaction means to show the difference between HIN and normal cells. We also used the R package scMLnet (v.0.1.0) to predict how the microenvironment regulated FOXP3 in Tregs (as described by Cheng et al.^40^).

### 2.14 Integration analysis of single‐cell data of invasive cancer cells and our data

The expression data of epithelial cells and invasive cancer cell data downloaded from GSE160269_(P1T‐E, P107T‐E, P16T‐E, P128T‐E, P130T‐E, P126T‐E and P127T‐E) were read and merged using Seurat R package. We then analysed the integrated data according to the steps described above.

### 2.15 Immunohistofluorescence detection

Formalin‐fixed paraffin‐embedded sections (3 mm) of human oesophageal epithelial tissues and organoids were detected by immunofluorescent staining with antibodies (Abcam) shown in Table S2. The sections were incubated with antibodies against KRT15 (1:200, ab80522; 1:50, ab52816), KRT19 (1:200, ab52625), STMN1 (1:2000, ab52630) and KRT13 (1:2000, ab16112). The sections were counterstained with DAPI (1:2000, G1012) to visualise the nuclei.

### 2.16 Primary cell culture

Fresh tissues from HIN (n = 3, by endoscopic dissection), adjacent normal tissues (n = 3, by biopsies) and invasive cancer (n = 3, by biopsies) were obtained from six patients. The tissues were washed with Sample Diluent (PreceDo Pharmaceuticals Co. Ltd., #03‐0001‐D.Rs), cut into small pieces (1–2 mm^3^) on ice and digested using enzymes (PreceDo Pharmaceuticals Co. Ltd., #02‐0001‐D.Es) according to the manufacturer's instructions. After filtration, the cell pellet was collected by centrifugation at 1500 rpm for 3 min at room temperature. The cell suspension was counted and assessed for viability using trypan blue staining with a haemocytometer. Next, the single‐cell suspension was cultured in Esophagus Carcinoma Cell Medium (PreceDo Pharmaceuticals Co. Ltd., #00‐F0006‐CM) with 3T3 cells (irradiated with γ rays [40 Gy]).

### 2.17 Karyotype analysis

Primary cells (as described above) were cultured at 37°C in a humidified atmosphere of 5% CO_2_. The culture was continued for 2–4 h after adding colchicine (Sangon Biotech, A600322‐0100), and the final concentration of colchicine reached 0.1–0.2 μg/mL. Cell division was halted at the metaphase stage of mitosis and chromosomal slides were prepared. Videotest–karyo software was used to perform karyotyping.

### 2.18 Organoid cultures

We employed the method described by Kijima et al.^41^ to generate 3D organoids. To ensure the tumour identity of HIN cells, we used primary cells with confirmed karyotypes (aneuploidy) to generate organoids (as described above). Cells were suspended in DMEM/F12 medium (Gibco, 11330‐032) and plated in Matrigel (Corning, 356231) on ice. After solidification at 37°C, Esophagus Carcinoma Organoid Medium (PreceDo Pharmaceuticals Co. Ltd., #00‐F0006‐OGM) was added to the culture at 37°C and replenished every 5 days.

### 2.19 Quantification of STMN1^high^ signal in oesophageal KRT15^high^ basal cells

Slides of normal oesophageal tissue (KRT15/STMN1 double‐stained) were recorded using the Pannoramic MIDI II (3DHISTECH Ltd). We manually distinguished between the papillary and interpapillary regions on three slides and obtained cell percentages. Then, a bar chart was generated by importing the cell percentages into GraphPad Prism (V 6.07).

### 2.20 Statistical analysis

Functions *t‐*test from R (V 4.0.3) was used to analyse tissue preference based on R_o/e_ values. *p* < 0.05 was considered statistically significant. Other statistical methods used are described in the figure legends.

## 3 RESULTS

### 3.1 A single‐cell transcriptome landscape of human oesophageal HIN

**FIGURE 1:**
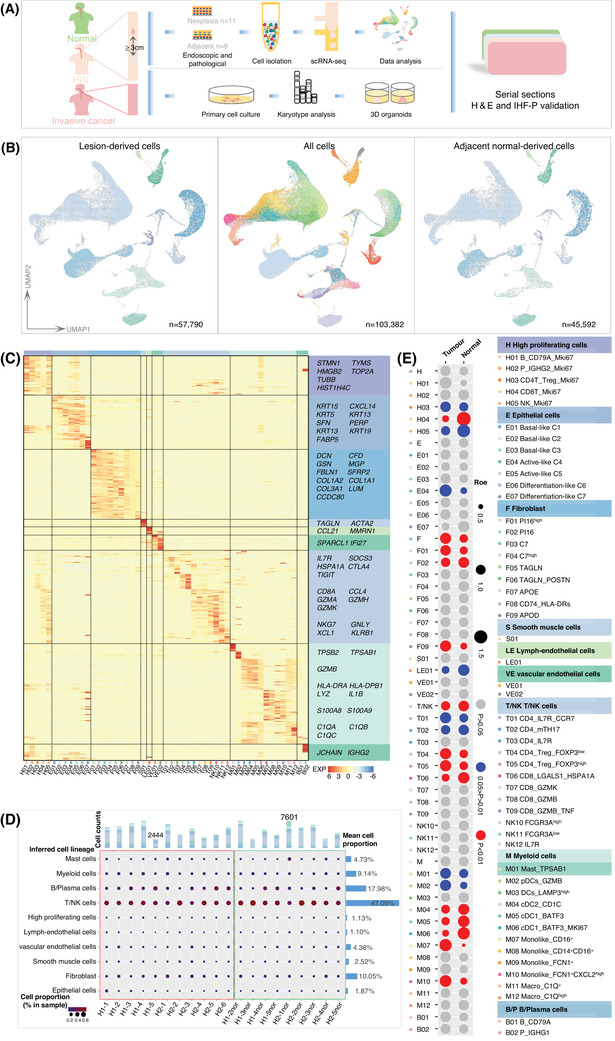
The features of human oesophageal HIN and adjacent normal tissues by single‐cell RNA sequencing. **(A**) Workflow shows experimental design and analysis. (**B**) Integral UMAP plots show all 51 defined clusters (middle), lesion‐derived cells (left) and adjacent normal‐derived cells (right). The text on the right panel shows the names of all clusters/subclusters. The major clusters were named after classical cell types. Subclusters were named after classic function markers or highly expressed genes. In case of multiple subclusters, another highly expressed gene was added to distinguish them. (**C**) Gene expression heatmap analysed by 10× scRNA‐seq. Subcluster numbers are indicated at the bottom. Representative TOP marker genes are labelled. Exp, Z‐score normalised mean expression. (**D**) The defined major cluster compositions in our data. The middle panel shows the defined major clusters (rows) by sample (columns). For each major cluster, the size and colour of the circle represent the proportion of cells in each individual sample. The magenta and green dashed boxes show HIN samples and adjacent normal samples, respectively. The histogram at the top shows the cell count for each sample. The samples with the most and the least cell counts are marked. The right histogram shows, for each specific major cluster, the mean cell proportion in the sample. The annotation at the bottom shows the corresponding sample IDs. (**E**) Tissue preference of each cluster/subcluster is measured by R_o/e_ values. For each specific cluster/subcluster, the size of the circle represents the mean R_o/e_ for samples (tumour or normal). *p*‐Values were calculated by a two‐tailed paired Student's *t*‐test (n = 18). For example, for the T04 subcluster, the mean R_o/e_ of the tumour samples was greater than that of the normal samples, and the *p* ≤ 0.01, indicating that the T04 subcluster was enriched in the tumour

To examine the cell populations and molecular features of HIN, 10× scRNA‐seq was performed on 20 endoscopic resections collected from 11 patients with HIN, including 11 HINs and 9 matched adjacent tissues (Figure 1A). Clinical and histopathological characteristics are summarised in the Supporting Information (Figure S1 and Table S1). Following quality filtering, 103 382 cells were retained for subsequent analysis, which detected a median of 1688 genes and 4963 UMIs per cell, similar to previous studies.^20^, ^42^

To define the cell population structure, all cells were first divided into 24 clusters and assigned to ten cell types based on canonical markers. These included epithelial cells (KRT5, KRT19 and SFN), fibroblasts (COL1A1, SFRP2, MMP2 and DCN), smooth muscle cells (ACTA2 and MYH11), vascular endothelial cells (PECAM1, ENG and PLVAP), lymphatic endothelial cells (PECAM1 and LYVE1), myeloid cells (CD68 and CD14), mast cells (CPA3 and TPSB2), B/plasma cells (CD79A and MS4A1) and T/NK cells (CD3D, CD3E and NKG7) (Figures S2A and S2C). Notably, different highly proliferating cell types (MKI67 and TOP2A) were clustered together. No histology‐specific populations were identified (Figure 1B, Figure S2B).

Compared with normal human tissue from the human cell landscape (HCL), which is a valuable and well‐annotated scRNA‐seq database, immune cells, particularly T/NK cells, were far more abundant. The proportion of immune cells in human HIN, about 80%, was notably higher than in the HIN/ESCC mouse model, where it was about 45%.^18^ Moreover, the B/plasma cell proportion in human HIN was significantly higher than in human ESCC, but there was no statistically significant difference between HIN and adjacent tissues (Figures 1D–1E, Figure S3).

We next performed unsupervised clustering for each cell type. Some clusters were defined based on initial results for their comparable enrichment (Figure 1E). A few subclusters were defined as ‘unknown’ for unexplained differentially expressed genes (DEGs). Overall, we defined 51 clusters, according to their canonical markers and specific signatures (Figure 1C). The cluster tissue preference was illustrated based on R_O/E_ ^36^ (Figure 1E). Some clusters, such as Treg cells (T04 and T05) and monocytes (M07 and M10), were lesion‐enriched; some others, such as dendritic cells (M04, M05 and M06) (Figure 1E), were normal‐enriched.

### 3.2 Heterogeneity of normal oesophageal BLCs

**FIGURE 2:**
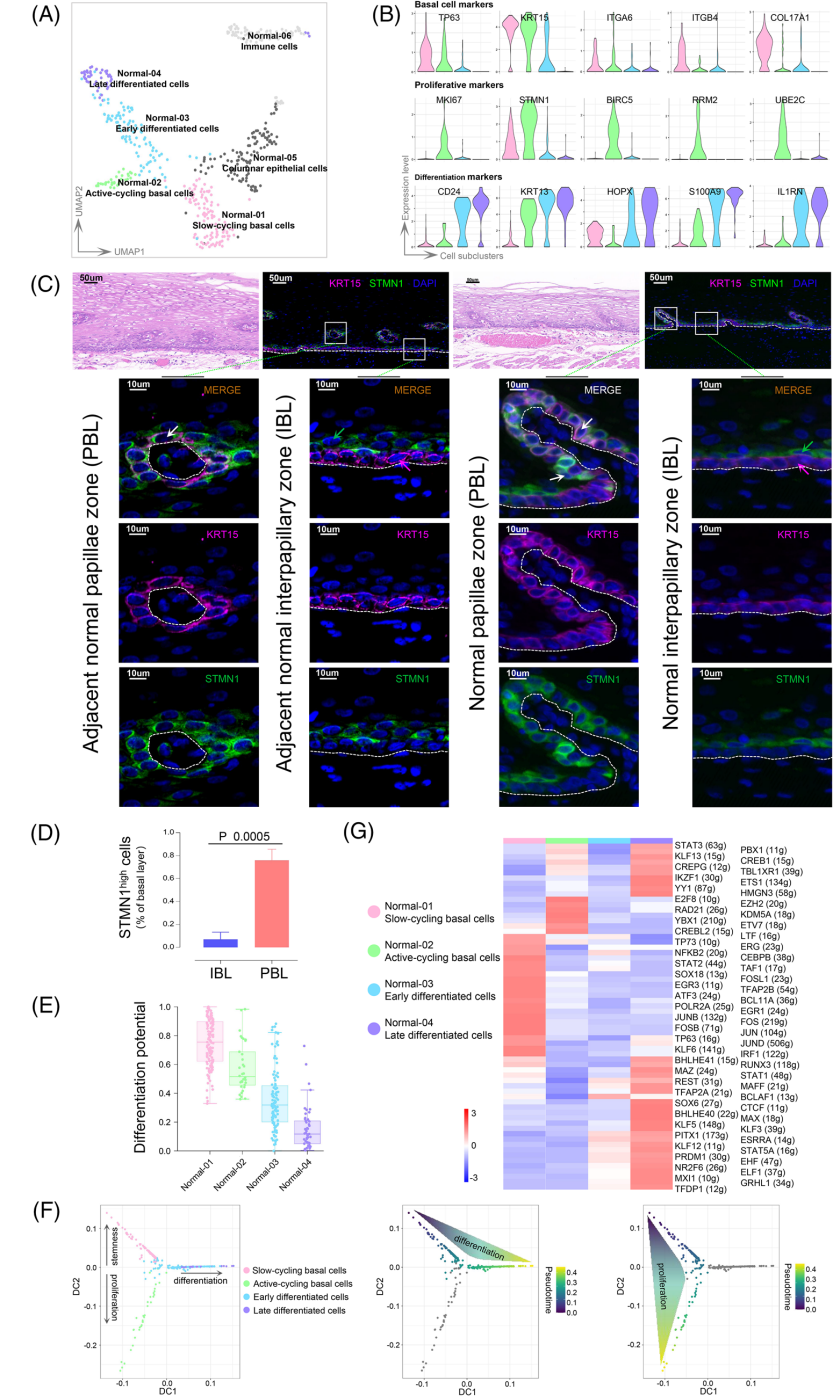
Heterogeneity of basal layer cells and differentiation dynamics of normal squamous epithelial cells. **(A**) UMAP plots indicate in different colours the cell types of the adjacent normal epithelial cluster. (**B**) Violin plots show the expression level of selected marker genes depicting the distinct epithelial cell types (indicated in different colours). (**C**) Immunofluorescence staining analysis of KRT15, KRT13 and STMN1 expression in human adjacent normal tissue and normal oesophageal epithelium. The white, magenta and green arrows indicate KRT15^high^STMN1^high^, KRT15^high^STMN1^low^ and KRT15^low^STMN1^high^ cells, respectively. Dashed lines: basement membrane. H&E, staining with hematoxylin and eosin. Scale bars, 50 and 10 μm for top and bottom panels, respectively. (**D**) Bar plots showing the proportion of STMN1^high^ cells in papillae (PBL) and interpapillary zone (IBL). *p*‐Value was calculated by unpaired *t*‐test. n = 3. (**E**) Differentiation potential of adjacent normal squamous epithelial cell types based on transcriptional diversity. For each box, the centre line, upper boundaries, lower boundaries and whiskers represent the median, 75th percentile, 25th percentile and 1.5 × interquartile range (IQR), respectively. (**F**) Diffusion map of adjacent normal squamous epithelial cells coloured by cell types (left), by differentiated cells pseudotime trajectory (middle), and by proliferative cells pseudotime trajectory (right). (**G**) Heatmap showing AUC scores of expression regulation by transcription factors of the indicated cell types, estimated by SCENIC

Studies in mouse models indicate that HIN originates from BLCs,^5^, ^7^, ^8^ but the heterogeneity of BLCs in mouse^9^, ^10^, ^11^, ^12^, ^13^ and human esophagus^11^, ^14^, ^15^, ^16^, ^17^ is controversial. To investigate the cellular origin of human HIN, we firstly focused on the cellular hierarchies of normal squamous epithelial cells. We identified six subclusters from the unsupervised clustering of epithelial cells from adjacent normal tissues (Figure 2A). Further, we defined four types of squamous epithelial cells, based on TOP DEGs and known markers. Normal‐01 carried high levels of basal markers, such as KRT15, TP63 and ITGB4, but low levels of proliferation‐related genes, indicating that they were slow‐cycling basal cells (SBCs). Normal‐02 was marked by cell‐cycle regulatory genes in addition to basal markers, such as STMN1, MKI67 and BIRC5, representing active‐cycling basal cells (ABCs). Normal‐03 and Normal‐04 represent early‐ and late‐differentiated cells, respectively, carrying different levels of differentiation‐related markers, such as CD24, KRT13 and HOPX (Figures 2A–2B). Normal‐02 co‐expressed the basal marker, KRT15, and mild levels of the early differentiation marker, KRT13, suggesting that these ABCs were committed to differentiation. Although Normal‐05 showed columnar glandular epithelial features, for example, KRT7, KRT8, KRT18 and so on (Figures S4A–S4B), it was extremely close to the basal cells (Figure 2A). Immunostaining showed that submucosal glandular epithelium express KRT19 but no KRT15 (Figures S4C–S4D), validating the glandular cell identity of Normal‐05. Normal‐06 was a separate cluster that possessed the characteristic of an immune population (Figure 2A, Figures S4A–S4B).

Next, we selected DEGs as markers and investigated their locations by immunostaining (Figure 2C, Figure S4E). We noted that KRT15^high^ cells were confined mainly to the basal layer but were different between the interpapillary basal layer (IBL) and papillae basal layer (PBL). IBL was dominated by KRT15^high^STMN1^low^ cells (Normal‐01), while PBL was populated with KRT15^high^STMN1^high^ cells (Normal‐02) (Figures 2C–2D). Notably, KRT15^high^STMN1^high^ cells were occasionally observed in the epibasal layer of IBL (Figure 2C), further supporting the notion that ABCs may commit to early differentiation. The data indicate that both the transcriptome and the location of BLC populations are heterogeneous.

We applied pseudotime analysis to investigate normal differentiation dynamics; normal differentiation is commonly blocked in tumours. Prior to this, an analysis based on differentiation potential (‘stemness’) was used to objectively determine differentiation origin (Figure 2E). SBCs showed the highest differentiation potential and were therefore placed at the root of the differentiation hierarchy^24^ (Figure 2F). This also indicated that KRT15^high^STMN1^low^ populations may be closer than other populations to stem or progenitor cells. Pseudotime trajectories showed that SBCs gave rise to early differentiated cells (Normal‐03), and then transitioned to late differentiated cells (Normal‐04). Like SBCs, ABCs had lower differentiation scores (DC1 scores) but bifurcated to attain their proliferative features. This differentiation trajectory was also supported by Monocle2 analysis (Figure S5). We also inferred TF activity (Figure 2G). As expected, differentiated cells showed high TF activities in differentiation‐associated genes, for example, SOX6, EHF and GRLH1. In SBCs, TP63 and the JUN/FOS family showed high activities.

### 3.3 Capturing tumour cells from squamous epithelial cell population

**FIGURE 3:**
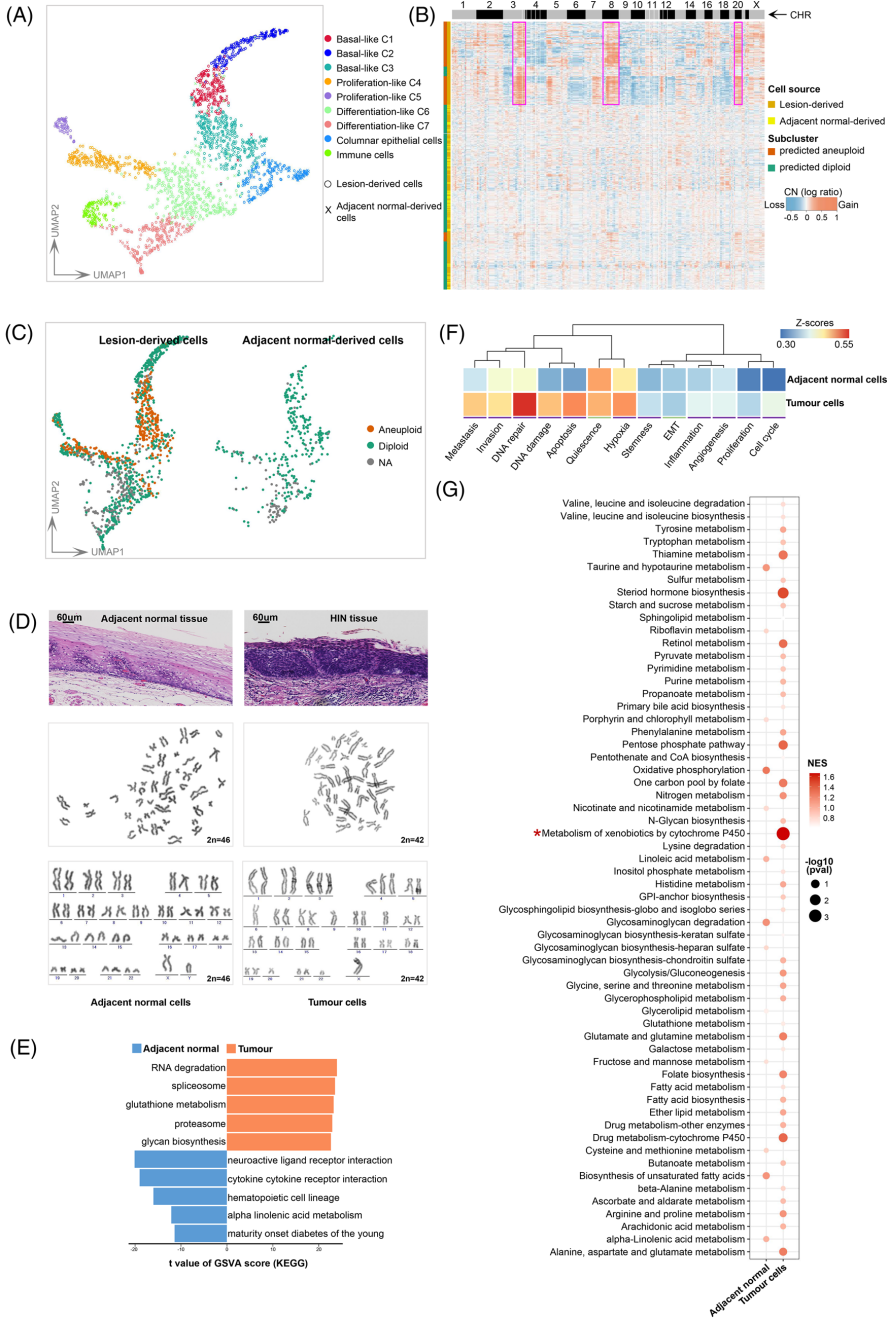
Identification of tumour cells from squamous epithelium. (A) UMAP plots indicate in different colours the subclusters of the epithelial cluster. The histopathological origin of cells is indicated in different shapes. (**B**) Clustered heatmap showing single‐cell copy number profiles estimated by CopyKAT from all squamous epithelial cells. CopyKAT classifications of diploid normal cells and aneuploid tumour cells and cell sources are indicated on the annotation bars to the left. Chromosome numbers are indicated at the top of the CHR bar. The magenta‐coloured boxes indicate chromosome amplification of 3q, 8q, 20p and 20q. (**C**) UMAP embedding of the inferred CopyKAT diploid and aneuploid copy number profiles (all squamous epithelial cells). NA: undefined cells. (**D**) Karyotype ideogram showing diploid cells in adjacent normal tissues and aneuploid cells in HIN tissues (n = 3). 2n: the number of chromosomes. H&E, staining with hematoxylin and eosin. Scale bars, 60 μm for top panels. (**E**) Differential pathway enriched in tumour cells and squamous epithelial cells derived from normal tissues by GSVA. Two‐sided unpaired limma‐moderated *t*‐test. (**F**) Heatmap showing different pathways enriched in adjacent normal and tumour cells by GSVA analysis, coloured by z‐score transformed mean GSVA scores. Different line colours indicate adjusted *p*‐values compared between two groups: purple, *p* < 0.05. Two‐sided unpaired limma‐moderated *t*‐test. (**G**) Bubble plot showing the enriched metabolic pathways between adjacent normal and tumour cells. *: the most significant pathway to metabolic heterogeneities

The unsupervised clustering of epithelial cells showed that cells derived from lesions and normal tissues showed similar clustering characteristics (Figure 3A, Figure S7A). Tumour tissue‐specific cells are common in invasive cancers, but no such population was found here, and tumour cells could not be identified based on clustering characteristics, as is done in other studies. To address this, we applied copyKAT,^29^ a novel unsupervised algorithm based on the identification of aneuploid copy number profiles, to our data. It successfully captured aneuploid tumour cells that showed obvious CNVs. The predicted tumour cells had frequent chromosome amplifications at 3q, 5p, 8q, 20p and 20q, which are commonly reported in human HIN and ESCC by bulk sequencing^1^, ^4^ (Figure 3B). No tumour cells were derived from adjacent normal tissues (Figure 3C), demonstrating the reliability of the result. Few predicted tumour cells were derived from late differentiated cells (Figure S7B), indicating that HIN differentiation had been blocked. We further applied primary cell culture combined with karyotype analysis to identify aneuploid cells. Aneuploid cells were not found in adjacent tissues but were ubiquitous in HIN tissues (Figure 3D). We also applied inferCNV to capture tumour cells and obtained similar results (Figures S6A–S6B). However, inferCNV defined more tumour cells and identified a few normal‐derived cells as tumorous (Figures S6B–S6C). Given that copyKAT is an unsupervised method that automatically designates reference cells, and more suitable for the analysis of data from newly developed high‐throughput scRNA‐seq platforms,^29^ we tended to adopt its result for subsequent analysis.

Differential analysis showed that proliferation and antigen presentation genes, such as STMN1 and HLA‐DRA, were slightly up‐regulated in tumour cells, whereas differentiation molecules, such as KRT13, HOPX and CD24, were highly expressed in normal cells (Figure S7C). Enrichment analysis showed that tumour cells were enriched for metabolism‐related pathways (Figure 3E). After a thorough evaluation of metabolic properties,^35^ we found that the metabolic activities of tumour cells were generally more active than normal cells. Glycolysis, a hallmark of cancer,^43^ was enriched, although not significantly, in tumour cells (Figure 3G). RNA splicing, a dysregulated process in mouse oesophageal^7^ and epidermal^44^ precancerous lesions, was also enriched in tumour cells. In addition, we applied CancerSEA,^32^ a rigorously screened and scRNA based cancer signature, to assess the functional states of the tumour cells. Tumour cells were more susceptible to hypoxia, DNA damage, DNA repair, invasion, metastasis and apoptosis activities (Figure 3F). These data suggest that there were indeed differences between HIN and normal cells at the single‐cell transcriptome level.

### 3.4 HIN originates from slow‐cycling basal cells and shows different degrees of differentiation

**FIGURE 4:**
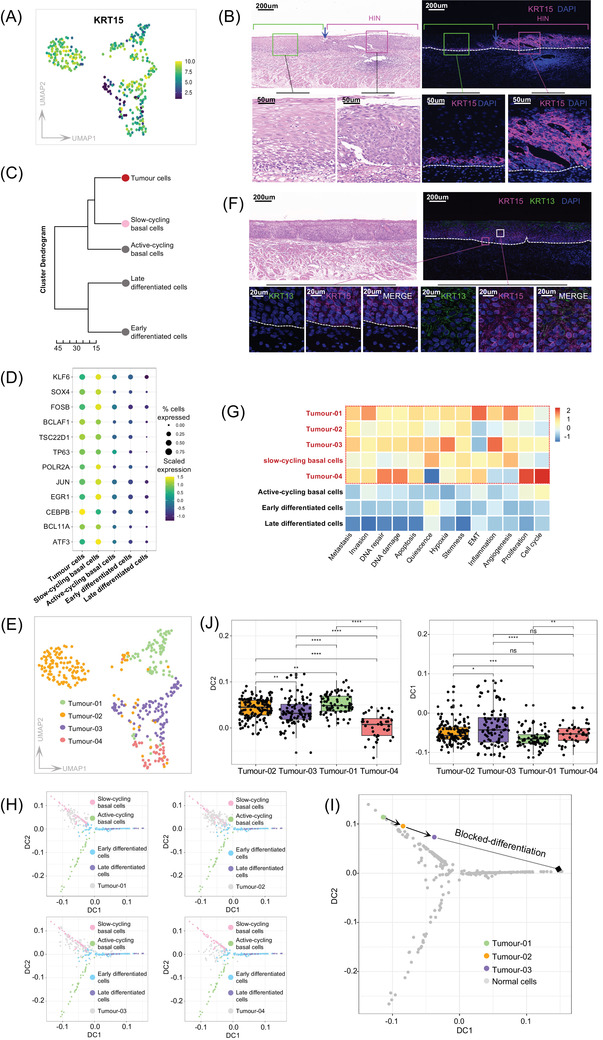
Reconstructing the lineage relations between tumour and normal populations. (A) UMAP plots showing the expression of basal layer cells marker KRT15 (yellow, high; black, low) in tumour cells. (**B**) Immunofluorescence staining analysis of KRT15 expression in human adjacent normal and HIN tumour tissue. Magenta and green square brackets indicate tumour and normal areas, respectively. Blue arrows indicating tumour boundaries. Dashed lines: basement membrane. H&E, staining with hematoxylin and eosin. Scale bars, 200 and 50 μm for top and bottom panels, respectively. (**C**) Similarity (Euclidean distance) between tumour cells and normal cell types based on all genes. (**D**) Bubble plot shows the expression of key TFs (identified in Figure 2) among tumour cells and normal cell types. The fraction of cells expressing TFs is indicated by the size of the circle, and their scaled expression levels are indicated by the colour of the circle. (**E**) UMAP plots show subclusters of tumour cells (indicated in different colours). (**F**) Immunofluorescence staining analysis of KRT15^+^KRT13^−^ (magenta box) and KRT15^+^KRT13^+^ (white box) cells in HIN tissue. Scale bars, 200 and 20 μm for top and bottom panels, respectively. Dashed lines: basement membrane. H&E, staining with hematoxylin and eosin. (**G**) Heatmap showing different pathways enriched in subclusters of tumour cells and normal cell types by GSVA analysis, coloured by z‐score transformed mean GSVA scores. (**H–I)** Diffusion maps of normal cells (coloured dots) with tumour subclusters (grey dots) projected onto the embedding (H) and mean diffusion components for tumour cells, coloured by tumour subclusters (I). Arrows and dotted lines indicate blocked differentiation of HIN. (**J**) Box plots show the diffusion components for all tumour cells by subcluster, DC2 (left); DC1 (right). For each box, the center line, upper boundaries, lower boundaries and whiskers represent the median, 75th percentile, 25th percentile and 1.5 × interquartile range (IQR), respectively. Two‐sided Wilcoxon rank‐sum test with *p* < 2.22 × 10^−16^ was considered statistically significant for any pairwise comparison

Studies in mouse models indicate that oesophageal HIN originates from BLCs.^5^, ^7^, ^8^ To test this hypothesis in humans, we first examined KRT15 expression (basal marker) in tumour cells and found that as predicted, KRT15 was highly expressed in tumour cells (Figures 4A–4B). Given the heterogeneity of BLCs in humans, we reconstructed the lineage relationships between tumours and normal populations. Hierarchical clustering showed the strongest similarities between tumour cells and SBCs (Figure 4C). Given the key role of TFs in cell‐fate decisions,^45^ we found that tumour cells showed the highest similarity to SBCs in TF expression (Figure 4D). To evaluate the lineage relationships of the potential heterogeneity of tumour cells, we reclustered tumour cells and obtained four populations (Figure 4E, Figure S9A). Compared with normal cell types, each tumour population showed the strongest similarity to SBCs across a broad set of cancer functional states^32^ (Figure 4G). These data suggested that HIN may arise from SBCs. To further explore their origin, we projected each tumour population onto pseudotime trajectories of normal cells, and found that they mapped primarily to SBCs (Figures 4H–4I). These data demonstrate the SBC identity of tumour cells from the global transcriptome, TFs, functional status and developmental pseudotime perspectives.

We noted that some tumour cells, for example, tumour‐03, carried early differentiation marker KRT13 (Figures S9A–S9B), which was validated in HIN tissues (Figure 4F, Figure S8). This indicated that tumour cells may have been in different differentiation gradients. Although tumour populations were mapped to SBC, we observed that tumour‐01 mapped closer to the beginning of the SBCs differentiation trajectory (Figures 4H–4I). Quantification of the differentiation and stemness scores confirmed these differences (Figure 4J). In addition, tumour‐01 showed stronger EMT and invasion, while tumour‐03 was enriched in differentiation‐related pathways (Figure S9D). Function analysis revealed that the greatest difference, such as antigen presentation pathways, was between tumour‐01 and tumour‐03 (Figures S9C and S9E). MHC II and antigen presentation pathways were enriched in tumour‐01 (Figures S9C and S9E). Metabolic assessment indicated that tumour cells were generally more active than their origin cells and tumour‐01 showed lower metabolic activities (Figure S9F). This observation was consistent with the observed differentiation gradients. Thus, the data demonstrated the differentiation heterogeneity of tumour cells.

### 3.5 HIN largely retains the transcriptional and functional features of the normal epithelium

**FIGURE 5:**
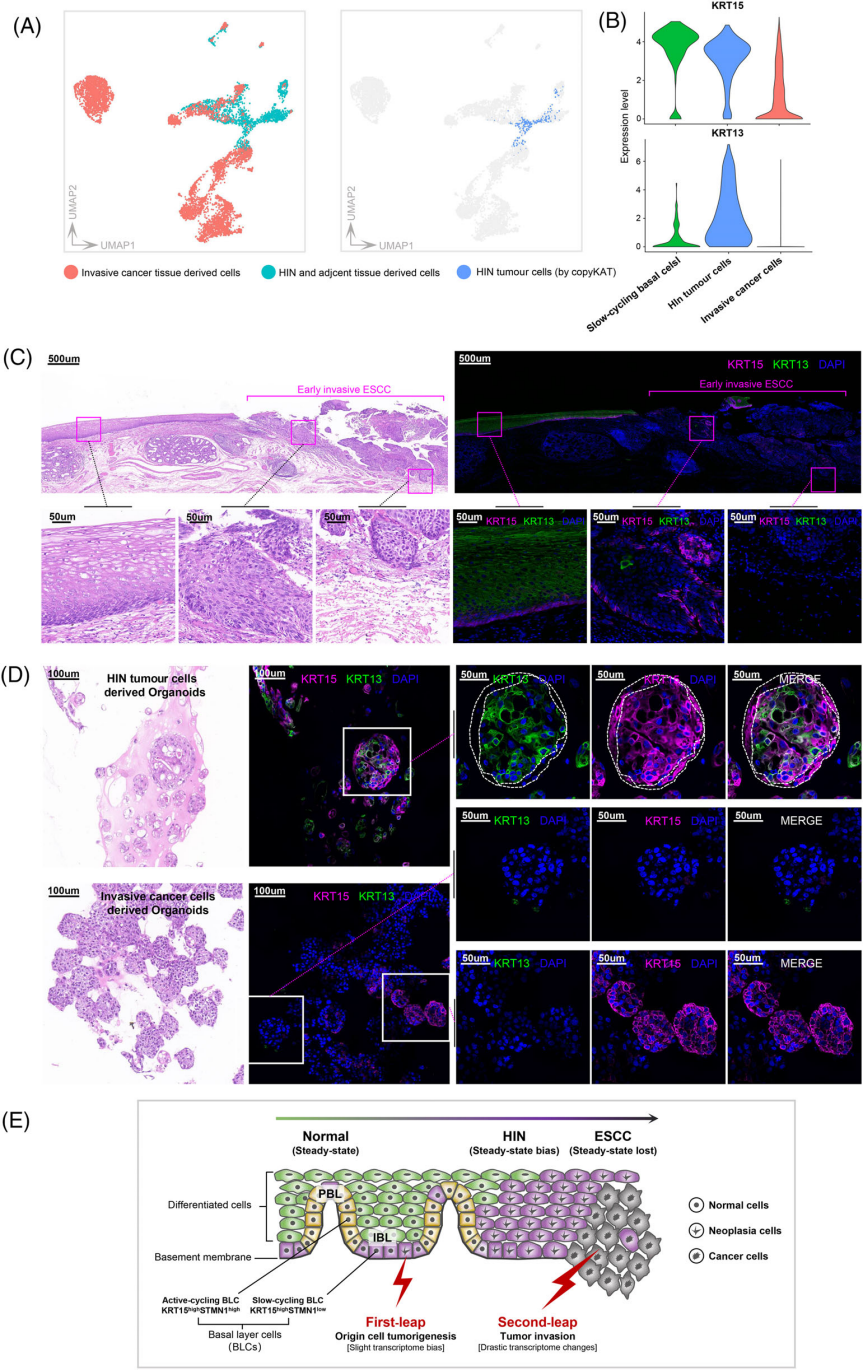
HIN partially retains the basal layer cell features and differentiation ability of normal epithelial. (A) UMAP plots show the heterogeneity between HIN cells and invasive cancer cells. (**B**) Violin plots show the expression of KRT15 (above) and KRT13 (below) among slow‐cycling cells, HIN tumour cells and invasive cancer cells. (**C**) Immunofluorescence staining analysis shows the expression of KRT15 and KRT13 in early invasive cancer. H&E, staining with hematoxylin and eosin. Magenta square brackets indicate early invasive cancer areas. Scale bars, 500 and 50 μm for top and bottom panels, respectively. (**D**) Immunofluorescence staining of oesophageal organoids for expression of KRT15 and KRT13. The dashed lines mark the range of differentiation phenotype cells (inner) and the size of organoid structures (outer). H&E, staining with hematoxylin and eosin. Scale bars, 100 and 50 μm for left and right panels, respectively. (**E**) Schematic illustration of the early development of human oesophageal HIN/ESCC. See text for details

Although the data suggested that HIN was indeed a tumour, we noted that its cells had clustering features similar to those of normal cells. This raises the question: when did the marked heterogeneity of invasive cancer emerge? In carcinogen‐induced HIN/ESCC models, pathologically specific epithelial populations were only present during the early invasive stage.^18^ To confirm this in humans, integration analysis was applied to our HIN data and published ESCC single‐cell data.^46^ Normal and HIN cells did indeed cluster together, but most ESCC cells were significantly farther away from them (Figure 5A). Previous studies suggested that normal epithelial features were absent at the invasive front of early ESCC invasions in mice.^6^ We found that the basal layer marker KRT15 and the differentiation marker KRT13 were absent in ESCC cells (Figure 5B); this was also observed in the bulk data (Figure S10). We further applied immunostaining to early invasive cancer tissues and found that the number of KRT13^+^ and KRT15^+^ cells had decreased (Figure 5C). The data indicate that the normal epithelial features retained in HIN may change drastically with tumour progression. To further elucidate these findings, we used 3D organoids to investigate their functional relevance. To ensure the tumour identity of HIN cells, we used the primary cells with confirmed aneuploidy to generate organoids. ESCC‐derived 3D structures showed severe cellular‐atypia and lacked differentiation. Under the same culture conditions, HIN‐derived 3D structures also lacked typical differentiation gradients, as described by Kijima et al.,^41^ indicating that they were neoplastic organoids (Figure 5D). Nonetheless, immunostaining showed that the differentiation phenotype (KRT13^+^) was present in all cells, except those in the peripheral layer, suggesting incomplete keratinisation (Figure 5D). These data indicate that HIN transcriptionally and functionally resembles normal epithelial cells, but may experience drastic changes during tumour invasion.

### 3.6 CD4^+^ T cells towards an immunosuppressive Treg phenotype

**FIGURE 6:**
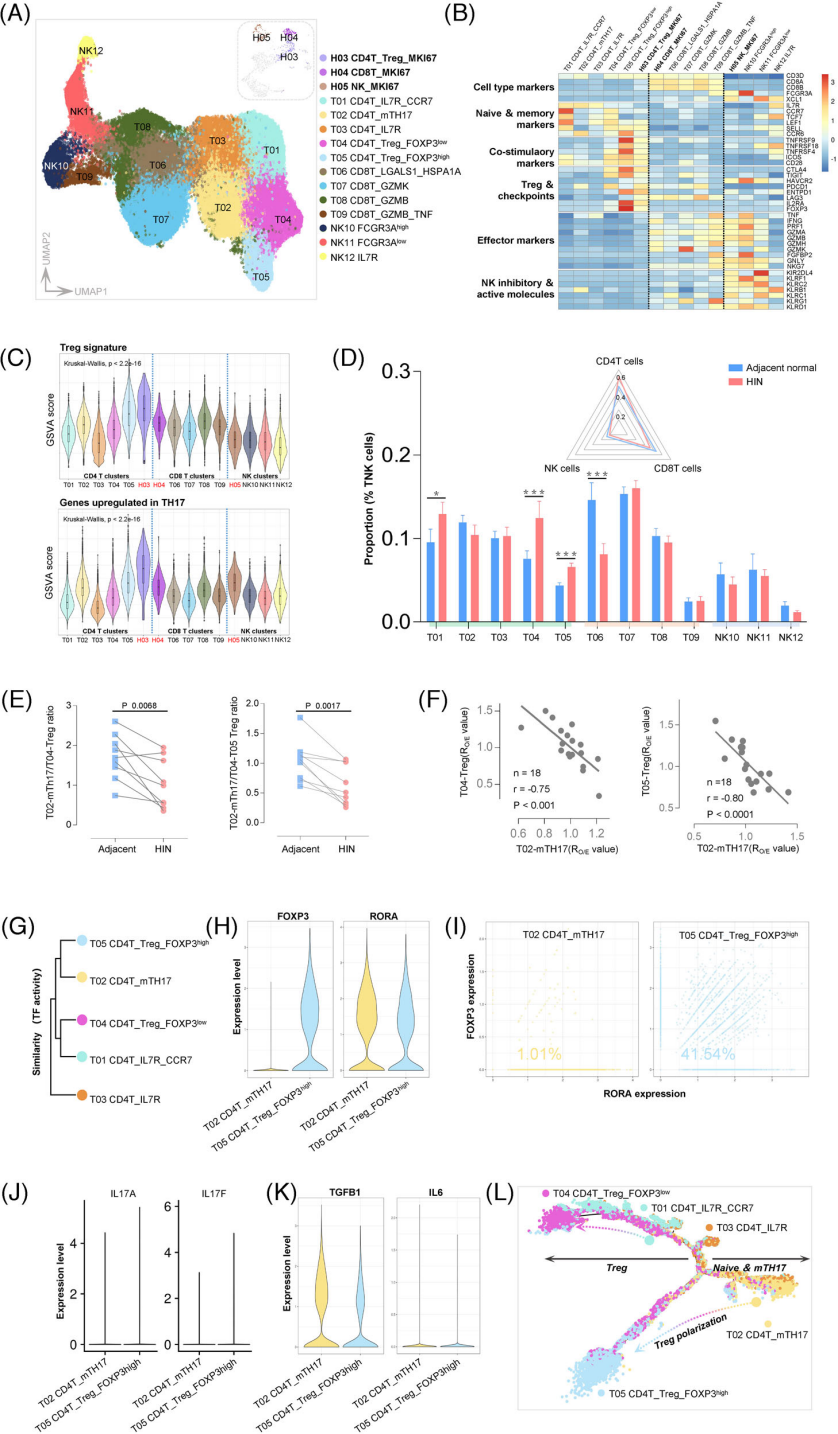
Features of TNK cells in human oesophageal HIN. (**A**) UMAP plots indicate different cell types of TNK cells in different colours. Embedded UMAP plots show TNK cells among highly proliferating cells. (**B**) Heatmap shows Z‐score normalised expression of selected T/NK cells function‐associated genes in each cell type. (**C**) Violin plots show the GSVA score of Treg (above) and TH17 (below) signature among different cell types of TNK cells. Box, median ± interquartile range. Whiskers, 1.5 × interquartile range. *p*‐Values across different cell types were calculated by a one‐way Kruskal–Wallis rank‐sum test. (**D**) Bar plots show the proportion of each cell type to total TNK cells between HIN (n = 9) and adjacent (n = 9) tissues. Each box represents the mean value, and whiskers indicate the standard error of the mean. Significant distribution differences are marked with asterisks based on *p*‐values of R_O/E_ (Figure 1). Embedded radar plots show the mean proportion of CD4^+^ T, CD8^+^ T and NK cells to total TNK cells between HIN and adjacent tissues. (**E**) The ratio of mTH17/Treg in HIN (n = 9) and adjacent (n = 9) tissues. *p*‐Values were calculated by two‐tailed paired Student's *t*‐test. (**F**) Correlation of tissue distribution (based on Ro/e values) between mTH17 and Treg (n = 18). Pearson correlation and linear regression. The *p*‐ and *r*‐values represent Pearson's correlation and its coefficient of determination. (**G**) Similarity among subclusters of CD4^+^ T cells based on TF activity (estimated by SCENIC). (**H**) Violin plots show the expression of FOXP3 and RORA in mTH17 cells and activated Treg cells. (**I**) Scatter plots show the frequency of mTH17 cells (left) or activated Treg cells (right) co‐expressing FOXP3 and RORA TFs. (**J–K**) Violin plots show the expression of selected genes in mTH17 cells and activated Treg cells. (**L**) The developmental trajectory of CD4^+^ T cells is inferred by Monocle2

Up to 50% of the Treg cells in our data were TNK cells (Figure 1D). We identified six CD4^+^ T, five CD8^+^ T and four NK subclusters (Figure 6A). Tissue enrichment of most CD4^+^ T clusters was significantly different between HIN and adjacent tissues, and CD4^+^ T subclusters showed the strongest costimulatory signature (Figures 6B and 6D, Figure S11). T01, T02 and T03 carried naive and memory signatures, for example, IL7R, CCR7 and TCF7, whereas T04, T05 and H03 expressed high levels of Tregs & checkpoints. T02‐mTH17 was defined as a memory IL17 helper cell for high expression of CCR6 and KLRB.^47^ T04‐Treg‐FOXP3^low^ and T05‐Treg‐FOXP3^high^, with varying expression of FOXP3, were termed resting and activated Treg cells, respectively.^48^ H03‐Treg‐MKI67 represents proliferating Tregs with high expression of FOXP3 and MKI67.

We noted that, in the same Treg cluster, costimulatory molecules were significantly higher in HINs than in adjacent tissues (Figure S13B). Compared with CD8^+^ T cells, the fraction of CD4^+^ T cells co‐expressed checkpoints was higher (Figure S12). This indicates that HIN cells may activate CD4^+^ T cells towards the Treg phenotype. Indeed, Tregs with the strongest Treg signature were also characterised by costimulatory signatures (Figure 6B, Figure S11), consistent with the role of costimulatory for maintaining Treg cells.^49^ Interestingly, we noted that Treg and TH17 signatures were related in Tregs and mTH17 (Figure 6C, Figure S14B). Given the TH17 response in carcinogen‐induced HIN/ESCC models,^18^ which may represent an acute inflammatory state, we investigated whether an imbalance of mTH17 and Treg cells existed in human HIN tumorigenesis. Firstly, we investigated the ratio of mTH17/Treg cells and identified an imbalance between the tumour and adjacent tissues (Figure 6E). We further noted that there was a strong negative correlation between the R_o/e_ values of mTH17 cells and Tregs (Figure 6F). T02‐mTH17 and T05‐Treg‐FOXP3^high^ further showed the highest TF activity similarity and the co‐expression of FOXP3‐RORA (Treg‐ and TH17‐specific differentiation‐related TFs^50^, ^51^) was higher in Tregs (Figures 6G–6I). While carrying RORA, T02‐mTH17 cells did not express canonical TH17 markers and high concentrations of TGFβ‐promoted Treg differentiation without IL6 (Figures 6J–6K). These results indicate that mTH17, the precursor of TH17 cells,^47^ tended to differentiate into Treg rather than the TH17 phenotype in human oesophageal HIN (Figure 6L).

Among CD8^+^ T cells, fully activated T09 cells account for only about 2% (Figure 6D, Figure S11), suggesting that cytotoxic immunity was insufficient. The age‐associated and exhausted‐like immune ageing hallmark,^52^ GZMK^high^ CD8^+^ T population (T07), was the most abundant CD8^+^ T cell (Figure 6D). This cell was sparse in mouse model^18^ and human ESCC.^20^ Given the increasing risk of HIN with ageing,^1^ we suspected that CD8^+^ T cells in HIN were mainly inactivated due to aging, rather than in an activation–exhaustion state, like in ESCC.^20^ Firstly, the number of proliferating CD8^+^ T (H04) was negligible, and naive CD8^+^ T cells were entirely absent (Figures 6A–6B), both of which are important features of immune ageing.^53^, ^54^

In HIN, the most exhausted population was proliferating CD8^+^ T cells. Immune populations in HIN were only moderately exhausted^55^ and there were no heavily exhausted CD8^+^ T cells, which are common in ESCC^20^ (Figure S11). Furthermore, the loss of costimulatory molecules was obvious in CD8^+^ T cells of HIN, signifying immune aging^56^ (Figure 6B, Figure S11). In contrast, the immune exhaustion hallmarks, that is, multiple co‐expressed checkpoints and costimulatory markers^56^ (e.g. CTLA4‐TIGIT and HAVCR2‐PDCD1 co‐expression representing severely exhausted state^57^) was obvious in CD8^+^ T cells of ESCC but inconspicuous in HIN (Figure 6B, Figure S12). Moreover, no significant difference of effector molecules was observed in CD8^+^ T cells between HIN and adjacent cells (Figure S13A). The data indicate that most CD8^+^ T cells in HIN are not fully activated, unlike in ESCC where these cells are activated but exhausted.

### 3.7 Loss of antigen presenting dendritic cells and gain of inflammatory monocytes in HIN

**FIGURE 7:**
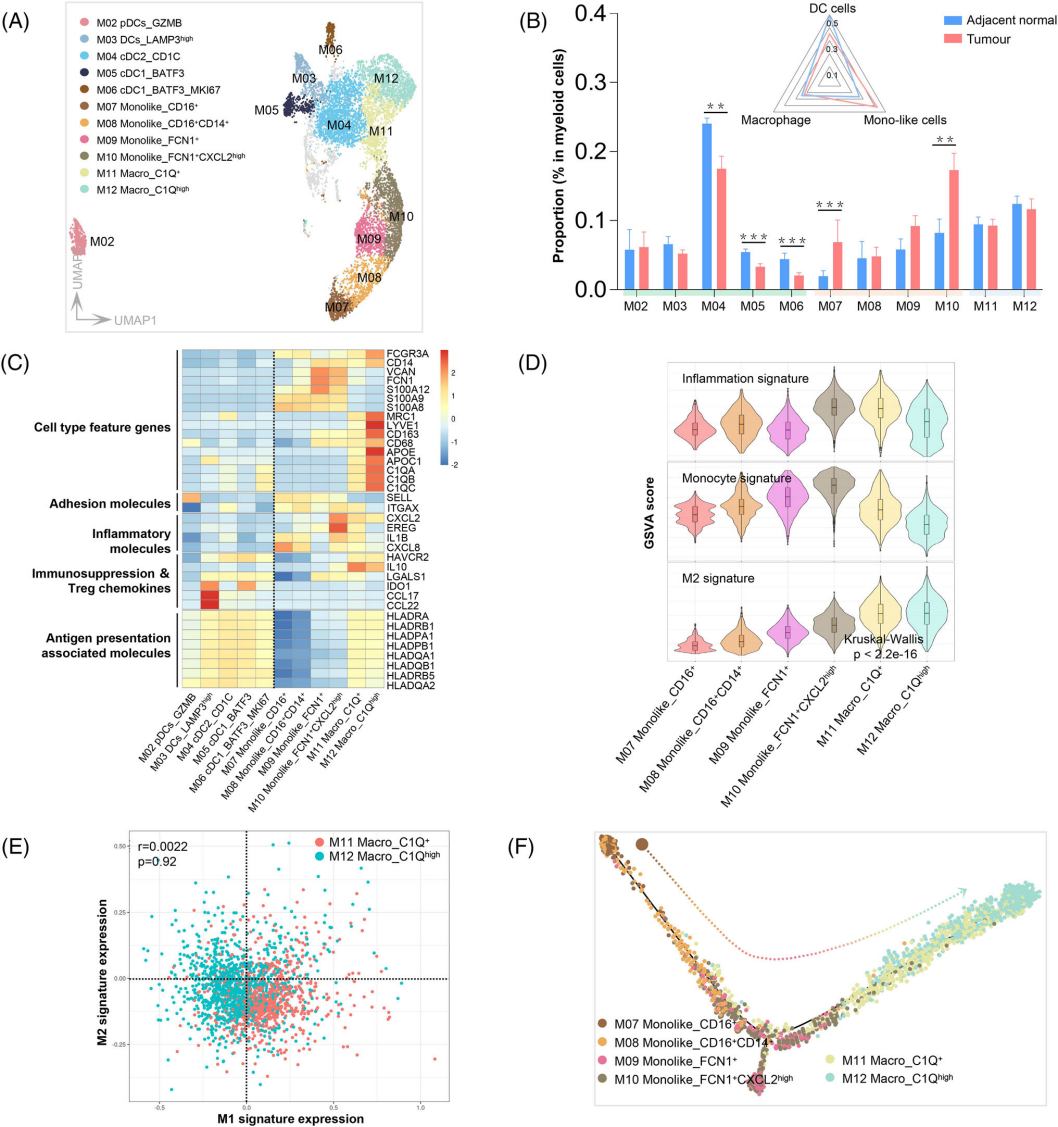
Characteristics of myeloid cells in human oesophageal HIN. (A) UMAP plots indicate the different types of myeloid cells in different colours. Unannotated cells are shown in grey. (**B**) Bar plots show the proportion of each cell type to total myeloid cells between HIN (n = 9) and adjacent (n = 9) tissues. Each box representes the mean value, and whiskers indicate the standard error of the mean. Significant distribution differences are marked with asterisks based on *p*‐values of R_O/E_ (Figure 1). Embedded radar plots show the mean proportion of dendritic cells, monocytes and macrophages to total myeloid cells between HIN and adjacent tissues. (**C**) Heatmap shows Z‐score normalised expression of function‐associated genes in each cell type among selected myeloid cells. (**D**) Violin plots show the GSVA score of selected signature gene sets among different cell types of monocytes/macrophages. Box, median ± interquartile range. Whiskers, 1.5 × interquartile range. *p*‐Values across different cell types were calculated by a one‐way Kruskal–Wallis rank‐sum test. (**E**) Scatter plots show the correlation between M1 and M2 signature per cell in macrophages; cells assigned to macrophages were coloured by cluster type. The *r*‐ and *p*‐values representing Pearson's correlation and its coefficient of determination. (**F**) The developmental trajectory of monocytes and macrophages inferred by Monocle2

The results stated above indicate that cytotoxic immunity was insufficient in HIN. Antigen‐presenting cells (APCs) involved lymphocyte activation prompted us to further investigate myeloid subclusters. We identified 12 subclusters: plasmacytoid DCs (GZMB), LAMP3^+^ DCs (LAMP3^high^), classical DC2 (CD1C), cDC1 (BATF3), proliferating cDC1 (MKI67), monocyte‐like cells (S100A8, S100A9, SELL) and macrophages (LYVE1, CD163, MRC1) (Figure 7A). Overall, cDCs were reduced in HIN (Figure 7B). Meanwhile, cDCs carried high levels of HAVCR2 and LGALS1, which inhibit the activation of CD8+ T cells and induce T cell apoptosis, respectively (Figure 7C). We also identified the novel LAMP3^high^ DCs^58^ in HIN, which occurred at a similar rate in both tumour and adjacent tissue, but were more abundant in ESCC^20^ (Figure 7B). The data indicate that the proportion of cDC decreased and the immunosuppression increased in HIN, supporting the suppression of CD8^+^ T cells.

Among monocytes/macrophages, M07 and M08 carried high levels of SELL and ITGAX, which were likely monocytes recently recruitment.^59^, ^60^ M10 was strongly inflamed; it carried high levels of inflammatory molecules and received the highest inflammation scores (Figures 7C–7D). M07 and M10 were abundant in tumour (Figure 7B), suggesting that HIN may continuously recruit monocytes and promote inflammation. Two macrophage clusters (M11 and M12), which showed comparable enrichment between tumour and adjacent tissues, were denoted as resident tissue macrophages. They carried high levels of MRC1, C1Q and IL10, a population similar to M2 cells. Unlike tumour‐associated macrophages in ESCC, in which M1 and M2 signatures were ambiguous, macrophages in HIN showed obvious M2 polarisation (Figures 7D–7E). Previous studies indicated that C1Q^+^ macrophages were connected to monocyte‐like cells^21^, ^61^ and our pseudotime analysis indicated same (Figure 7F).

In addition, we investigated the contribution of fibroblasts and cell–cell communications to the microenvironment. The major subclusters (F01–F06) of fibroblasts secreted large amounts of chemokines, cytokines and growth factors such as CCL‐chemokine ligand 19 (CCL19), CXCL‐chemokine ligand 12 (CXCL12) and insulin‐like growth factor binding proteins (IGFBPs) (Figure S15). The tumour‐enriched cluster F01 highly expresses PI16 which enhances the transendothelial migration of monocytes.^62^ To investigate potential cell–cell communications, we focused on subclusters that are significantly varied in HIN and are strongly correlated with each other (Figure S16A). Compared between HINs and SBCs, several significantly altered ligand–receptor pairs were identified. The interaction of CD58‐CD2 and LTBR‐LTB with HIN cells and Tregs had a higher interaction score, and HIN cells expressed high levels of CD58 and LTBR (Figures S16B–S16C). Previous studies have reported that the CD58‐CD2 interaction plays a key role in activating and maintaining Tregs.^63^, ^64^ Considering the direct role of FOXP3 in Tregs, we used scMLnet^40^ to predict how the microenvironment regulated FOXP3 in Tregs. The Multilayer Signaling Network revealed that, in addition to HIN, fibroblasts enriched in tumours also regulate FOXP3 expression (Figure S16D). This suggests that HIN cells and stromal cells may work together to promote and maintain the inflammatory and immunosuppressive microenvironment.

## 4 DISCUSSION

The molecular and functional properties of tumours are determined by intrinsic origin cells and the extrinsic micro‐environment. In this study, we present a comprehensive depiction of the single‐cell transcriptome landscape of human oesophageal HIN. We identify the heterogeneity of oesophageal BLCs, by transcriptome and location in humans. Further, we present evidence indicating that human HIN may originate from slow‐cycling KRT15^high^STMN1^low^ BLCs. We also show that HIN generally retains the BLC features and differentiation ability of the normal epithelium, which may change dramatically in the tumour invasion stage. These multistep changes in the transcriptome are helpful in understanding HIN initiation and early development. Immunosuppressed CD4^+^ T subsets, inactivated CD8^+^ T subsets and inflammatory monocyte and fibroblast subsets are the predominant features of the unique micro‐environment of human HIN. This is the first comprehensive and in‐depth genomic investigation of human oesophageal HIN and these findings provide insight into the cellular and molecular aspects of tumour origination and early development.

Previous studies on the origin of oesophageal HIN were mainly conducted in transgenic mice. In Krt5‐CreER mice, Sox2 overexpression resulted in hyperplasia or ESCC in the forestomach.^8^ Similar results were observed in another Krt5‐CreER/Krt15‐CrePR mouse model.^5^ These studies reveal that HIN or ESCC originate from Krt5^+^/Krt15^+^ BLCs in mice. In our analysis of human oesophageal squamous epithelial cells, scRNA‐Seq also sorted out human BLCs marked with KRT15 but clustered them into two subpopulations according to the expression level of the proliferation‐related gene STMN1: the slow‐cycling KRT15^high^STMN1^low^ subset and the active‐cycling KRT15^high^STMN1^high^ subset. This indicates that there are significant differences in the proliferative activity of human oesophageal BLCs. This has also been observed in single‐cell data from normal human oesophagus, but the distribution heterogeneity of BLCs has not been fully verified in this research.^14^ Compared with other studies, which are divided on whether cycling BLCs are present in the IBL^15^ or the PBL,^17^ our data show that active‐cycling BLCs are mainly located in PBL. As the first experimental evidence to demonstrate bioinformatics predictions, our research is not only indispensable for exploring tumorigenesis but also helpful in exploring normal epithelial homeostasis and wound healing.

Although HIN tumour cells were heterogeneous (with different differentiation degrees), transcriptional similarity strongly suggested that they originated from KRT15^high^STMN1^low^ BLCs. Earlier studies have shown that BLCs in PBL contain more IdU label‐retaining cells,^16^ but the retention seems shorter than that in IBL.^16^ This indicates that ABCs may be weaker than SBCs in terms of stemness or quiescence, and may commit to early differentiation. This finding has also been supported by our data. As a result, tumorigenic mutations and genetic alterations are less likely to persist in ABCs. In addition, our cohort lacked HIN tissues dominated by the differentiation phenotype of tumour cells. These findings suggest that ABCs are unlikely to be the cellular origin of human oesophageal HIN.

Contrary to our expectations, unsupervised clustering did not cluster tumour cells separately from squamous epithelial cells. This raises the question: when did the marked heterogeneity of invasive cancer emerge? While this study was not designed to address this question, revealing it during tumour early development would strengthen the evidence of tumour origin. Our evidence suggests that the drastic transcriptome changes may occur during the tumour invasion but not during the HIN stage. A 4‐nitroquinoline 1‐oxide induced mouse HIN/ESCC model shows that loss of normal epithelial characteristics appears at the tumour invasive front in early oesophageal invasive cancer.^6^ Another sorafenib induced mouse model found that the cell behaviour of neoplastic and surrounding cells was convergent but changed dramatically during tumour invasion.^7^ This has also been observed in the multistep processes of ESCC development induced by carcinogens,^18^ in which pathology‐specific epithelial populations are only present in the early invasive stage.^18^ Thus, the existing literature and our data suggest that the drastic changes in the transcriptome may emerge during tumour invasion, and not during the HIN stage. Based on this, we define two leaps during early ESCC development in humans (Figure 5E): ‘Tumorigenesis’ and ‘Drastic transcriptome changes’.

During the HIN stage, although the tumour identity of HIN is clear, cells may establish a new steady state to maintain epithelium homeostasis.^7^ This could be also supported by the rapid turnover of normal and HIN cells (one to two times per week^7^, ^13^) and the fact that HIN takes years to develop into ESCC. The second leap may emerge during tumour invasion. The definition of two leaps implies that there are many opportunities for intervention in the HIN stage. In addition, it suggests to us that what we know about invasive cancer may not be fully applicable to HIN, for example, caution is warranted where markers of oesophageal invasive cancer are applied for early diagnosis.

The inflammation induced by acid stress^5^ and Stat3 activation^8^ promoted tumorigenesis in the mouse forestomach, demonstrating the role of the microenvironment in HIN initiation and progression. In human HIN, activated CD4^+^ Tregs and not fully activated CD8^+^ T cells help form immunosuppressive surroundings. We did not find effector CD4^+^ clusters in human HIN, but the presence of mTH17 cells suggested that the TH17 response may occur during the acute first‐leap stage of, which was observed in the carcinogen‐induced HIN model.^18^ Compared with CD4^+^ T cells, the most abundant CD8^+^ T cells were GZMK^+^ CD8^+^ T cells, which is an immune aging hallmark.^52^ Unlike in HIN, heavily exhausted CD8^+^ T clusters in ESCC co‐express high levels of multiple checkpoints, effector molecules and costimulatory markers.^20^ This indicates that they have fought or are fighting cancer cells. It also suggests that the second leap of HIN may be accompanied by the reactivation of cytotoxic CD8^+^ T cells, but it may be already too late. Therefore, the reactivation of cytotoxic CD8^+^ T cells during the HIN stage may be a strategy to inhibit HIN initiation and progression. Previous studies have shown that immunosuppressive Tregs may downregulate lymphocyte activation and immune evasion in cancer^65^ and substantial change of cDCs may also lead to insufficient cytotoxic CD8^+^ T cells.^66^ Accordingly, our findings suggest that a variety of cellular treatment approaches to enhance functions of cytotoxic T cells during the HIN stage.

However, it should be noted that an immune active monocyte subset promoting immune protection and exhibiting pro‐inflammatory effects was also enriched in the HIN micro‐environment. For HIN, this microenvironment may be a paradox: Tregs may be indispensable for excessive inflammation, but sustained immunosuppression may promote the immune escape of HIN cells, leading to the second leap. This highlights the complexity of immune regulation and the importance of the fine balance between stimulatory and inhibitory immune signals to determine the outcome of HIN.

There are certain limitations to our study. Due to a significant increase in immune cells, our data for HIN cells were based on a relatively small number of epithelial cells. Although cell sorting may increase the proportion of epithelial cells, to ensure the accuracy of clinicopathological diagnosis, we could only obtain limited human HIN tissues, which were not adequate for such sorting and sequencing. This may have resulted in some loss of cellular and molecular information during scRNA‐Seq. In addition, transcriptional similarity is not the same as causality. Although lineage tracing is a gold standard in tracking cell fate and has been successfully applied in model animals to investigate tumour origin, it is not feasible in humans.

In summary, our study provided a comprehensive single‐cell transcriptome landscape of human oesophageal HIN, identifying which population of cells can give rise to HIN and the unique microenvironment characteristics of HIN. These findings pave the way for cancer prevention strategies that would be able to block tumour progression and even prevent cancer initiation.

## CONFLICT OF INTEREST

The authors declare no conflict of interest.

## Supporting information


**
Figure S1. H&E staining of 11 HIN tumour tissues.** H&E staining of 11 HIN specimens for sequencing, showed the 9.0x and 40X, respectively. Representative field from each specimen was shown (40X)
**Figure S2. Clustering characteristics of all cells**. A. UMAP plots show cells coloured by 24 clusters (original by Seurat) (left), and 10 cell types (defined) (right). B. UMAP plots show all clusters (Big panel: Coloured by patient IDs). Split UMAP plots show all clusters by single sample (Small panels: Coloured by cell types). C. UMAP plots show the expression levels of canonical marker genes for 10 cell types. Epithelial cells (KRT5, KRT19 and SFN), fibroblast (COL1A1, SFRP2 and MMP2, DCN), smooth muscle cells (ACTA2 and MYH11), vascular endothelial cells (PECAM1, ENG and PLVAP), lymphatic endothelial cells (PECAM1 and LYVE1), myeloid cells (CD68 and CD14), mast cells (CPA3 and TPSB2), B/plasma cells (CD79A and MS4A1), T/NK cells (CD3D, CD3E and NKG7) and high proliferating cells (MKI67 and TOP2A)
**Figure S3. Comparison of major cell types with other databases**. Radar plots show cell proportion in human oesophageal HIN compared with (A) human cell landscape (HCL), (B) human invasive oesophageal cancer (ESCC) and (C) a carcinogen induced HIN/ESCC mouse model
**Figure S4. Characteristics of non‐squamous epithelial subclusters and the heterogeneity of basal layer cells**. A. Heatmap of Z‐score normalised expression of TOP differential expression genes (DEGs) in subcluster of epithelial cluster. B. Violin plots show the expression level of selected marker genes indicating of the distinct cell types (indicated in different colours). C. Violin plots show the expression of KRT15 and KRT19 in slow‐cycling basal cells (Normal‐01) and columnar epithelial cells (Normal‐05). D. Immunofluorescence staining analysis of KRT15 and KRT19 expression in human adjacent normal oesophageal epithelium. The arrows indicate submucosal gland. Scale bars, 200 and 20 μm for top and bottom panels, respectively. H&E, staining with hematoxylin and eosin. E. Immunofluorescence staining analysis of KRT13, and STMN1 expression in human adjacent normal and normal oesophageal epithelium. The white, magenta and orange arrows indicate KRT13^high^STMN1^high^, KRT13^high^STMN1^low^ and KRT13^low^STMN1^low^ cells, respectively. The dashed lines indicate the basement membrane. Scale bars, 50 and 10 μm for top and bottom panels, respectively. H&E, staining with hematoxylin and eosin
**Figure S5. Developmental trajectory inferred by Monocle 2**. Developmental trajectory of adjacent normal squamous epithelial cells inferred by Monocle 2
**Figure S6. Capturing tumour cells by inferCNV**. A. Clustered heatmap show single‐cell copy number profiles estimated by inferCNV from all squamous epithelial cells. Magenta boxes indicate chromosome amplifications of 3q, 8q, 20p and 20q in some lesion‐derived cells. Chromosome numbers are indicated at the bottom. B. InferCNV classifications of normal cells and tumour cells are indicated on the annotation bars to the left. A few normal‐derived cells were identified as tumour cells. Chromosome numbers are indicated at the bottom bar. C. Comparison of tumour cells captured by copyKAT and inferCNV.
**Figure S7. Comparison of tumour cells and normal cells**. A. Heatmap of Z‐score normalised expression of TOP 10 differential expression genes (DEGs) in each subcluster of epithelial cluster. B. Bar plot shows the fraction of tumour cells identified by CopyKAT in each subcluster (from all squamous epithelial cells). C. Volcano plot shows DEGs in tumour cells identified by CopyKAT in comparison with all squamous epithelial cells derived from normal tissues. Representative genes are labelled.
**Figure S8. Investigating differentiation phenotype cells in HIN tissues**. Immunofluorescence staining analysis of KRT15^+^KRT13^−^ (magenta arrows) and KRT15^+^KRT13^+^ (white arrows) cells in HIN tissue. The dashed lines indicate the basement membrane. Scale bars: 60 μm. H&E, staining with haematoxylin and eosin
**Figure S9. Characteristics of tumour cell subclusters**. A. Heatmap shows the relative expression level of TOP10 DEGs across tumour cells, sorted by subclusters. B. UMAP plots shows the expression of differentiation marker genes KRT13 (yellow, high; black, low). C. Heatmap shows different pathways enriched in subclusters of HIN cells by GSVA analysis, coloured by z‐score transformed mean GSVA scores. Including GO‐BP (n = 7481) and KEGG (n = 186) gene sets. Only the pathways (rows) that were differentially expressed across different subclusters are shown. The selected pathways are indicated. D. Representative violin plots of selected pathways that showed significant differences between Tumor‐01 and Tumor‐03. Box, median ± interquartile range. Whiskers, 1.5 × interquartile range. *p*‐Values were calculated by a two‐sided Wilcoxon rank‐sum test. E. Violin plots show the expression level of selected MHC II & Antigen presentation related genes between Tumor‐01 and Tumor‐03 (indicated in different colours). F. Bubble plot shows the enriched metabolic pathways between slow‐cycling basal cells and tumour cells (left), and among subclusters of tumour cells (right). Red arrow indicates the significant pathway (|NES| > 1 and GSEA *p*‐value < 0.05) to the metabolic heterogeneities.
**Figure S10. Investigating the expression of KRT15 and KRT13 in bulk data**. Bar plots show the median expression of KRT15 (left) and KRT13 (right) in invasive oesophageal cancer and paired normal tissue. Data from GEPIA: http://gepia.cancer‐pku.cn/index.html
**Figure S11. Comparison of function state of TNK populations**. Violin plots show the GSVA score of selected signature gene sets among different cell types of TNK cells. Box, median ± interquartile range. Whiskers, 1.5 × interquartile range. *p*‐Values across different cell types were calculated by a one‐way Kruskal–Wallis rank‐sum test.
**Figure S12. The co‐expression of checkpoints in T cells**. Scatter plots show the percentage of CD8 T cells (A) or CD4 T cells (B) coexpressing HAVCR2‐PDCD1 (left) or GIGHT‐CTLA4 (right) checkpoints
**Figure S13. Comparison of the function gene expression between HIN and adjacent normal tissue in same cell cluster**. Violin plots show the expression of selected effector genes in CD8 T cells (A) and co‐stimulatory genes in activated Treg cells (B) between HIN and adjacent normal. *p*‐Values from pairwise comparisons are calculated using Wilcoxon rank sum test.
**Figure S14. The correlation of function gene sets in selected cell types**. A. Scatter plots show the correlation between the co‐stimulatory and Treg scores in rest Tregs (left) and activated Tregs (right). The *r*‐ and *p*‐values represent Pearson's correlation and its coefficient of determination. B. Scatter plots show the correlation between the TH17 scores and Treg scores in memory TH17 (left), rest Tregs (middle) and activated Tregs (right). The *r*‐ and *p*‐values represent Pearson's correlation and its coefficient of determination.
**Figure S15. Inflammatory and immunosuppressive fibroblasts in the microenvironment of HIN**. A. UMAP plots show cell types (indicated in different colours) of fibroblasts. Unannotated cells are shown in grey. B. Bar plots shows the proportion of each cell type to total fibroblasts between HIN (n = 9) and adjacent (n = 9) tissues. Each box represented the mean value, and whiskers indicate the standard error of the mean. The significant distribution differences are marked with asterisks based on *p*‐values of R_O/E_ (Figure S1). C. Heatmap shows Z‐score normalised expression of selected genes in each subcluster of fibroblasts. D. Violin plots show the expression level of LGALS1 in main cell populations (indicated in different colours).
**Figure S16. Enriched ligand–receptor cell–cell communication networks between HIN cells (or slow‐cycling cells) and focused immune cell subpopulations**. A. Heatmap shows the correlation of R_O/E_ values among subclusters. B. The significant ligand–receptor pairs (*p* < 0.05) between selected cell clusters and HIN cells (or slow‐cycling basal cells). The Interaction (difference value) = |Interaction mean value in HIN cells – Interaction mean value in slow‐cycling basal cells|, and the colour gradient indicates the mean level of interaction. Green plots represent the *p* value of ligand–receptor interaction was less than 0.05 in HIN cells or slow‐cycling basal cells: for example, the *p*‐value of LTBR‐LTB interaction is less than 0.05 between T04 and HIN cells, but it is greater than 0.05 between T04 and slow‐cycling basal cells. The means of the average expression levels of interacting molecule 1 in cluster 1 and interacting molecule 2 in cluster 2 are indicated by colour. C. Violin plots show the expression level of LTBR and CD58 between HIN cells and slow‐cycling basal cells (indicated in different colours). D. Multilayer networks produced by scMLnet depicting regulation of FOXP3 in Tregs (T04) by other types of cells. The multilayer signalling network consists of four layers: ligand layer, receptor layer, TF layer and target gene layer (i.e. FOXP3). The four layers constitute the inter‐/intra‐cellular signalling network: ligand–receptor subnetwork, receptor–TFs subnetwork and TFs–target gene subnetwork

Table S1 Clinicopathological information of samples for single‐cell RNA‐seq in this study

Table S2 Immunofluorescent antibodies used in this study
