# Supplementary material for: Single‐cell transcriptomics provides insights into the origin and microenvironment of human oesophageal high‐grade intraepithelial neoplasia
Source: Clin Transl Med. 2022 May 24;12(5):e874. doi: 10.1002/ctm2.874 (PMC9128161; doi:10.1002/ctm2.874)
Supplement: Supplementary file 1 — Figure S1. H&E staining of 11 HIN tumour tissues. H&E staining of 11 HIN specimens for sequencing, showed the 9.0x and 40X, respectively. Representative field from each specimen was shown (40X) Figure S2. Clustering characteristics of all cells. A. UMAP plots show cells coloured by 24 clusters (original by Seurat) (left), and 10 cell types (defined) (right). B. UMAP plots show all clusters (Big panel: Coloured by patient IDs). Split UMAP plots show all clusters by single sample (Small panels: Coloured by cell types). C. UMAP plots show the expression levels of canonical marker genes for 10 cell types. Epithelial cells (KRT5, KRT19 and SFN), fibroblast (COL1A1, SFRP2 and MMP2, DCN), smooth muscle cells (ACTA2 and MYH11), vascular endothelial cells (PECAM1, ENG and PLVAP), lymphatic endothelial cells (PECAM1 and LYVE1), myeloid cells (CD68 and CD14), mast cells (CPA3 and TPSB2), B/plasma cells (CD79A and MS4A1), T/NK cells (CD3D, CD3E and NKG7) and high proliferating cells (MKI67 and TOP2A) Figure S3. Comparison of major cell types with other databases. Radar plots show cell proportion in human oesophageal HIN compared with (A) human cell landscape (HCL), (B) human invasive oesophageal cancer (ESCC) and (C) a carcinogen induced HIN/ESCC mouse model Figure S4. Characteristics of non‐squamous epithelial subclusters and the heterogeneity of basal layer cells. A. Heatmap of Z‐score normalised expression of TOP differential expression genes (DEGs) in subcluster of epithelial cluster. B. Violin plots show the expression level of selected marker genes indicating of the distinct cell types (indicated in different colours). C. Violin plots show the expression of KRT15 and KRT19 in slow‐cycling basal cells (Normal‐01) and columnar epithelial cells (Normal‐05). D. Immunofluorescence staining analysis of KRT15 and KRT19 expression in human adjacent normal oesophageal epithelium. The arrows indicate submucosal gland. Scale bars, 200 and 20 μm for top and bottom panels, respectivel [file CTM2-12-e874-s003.pdf]

Figure S1

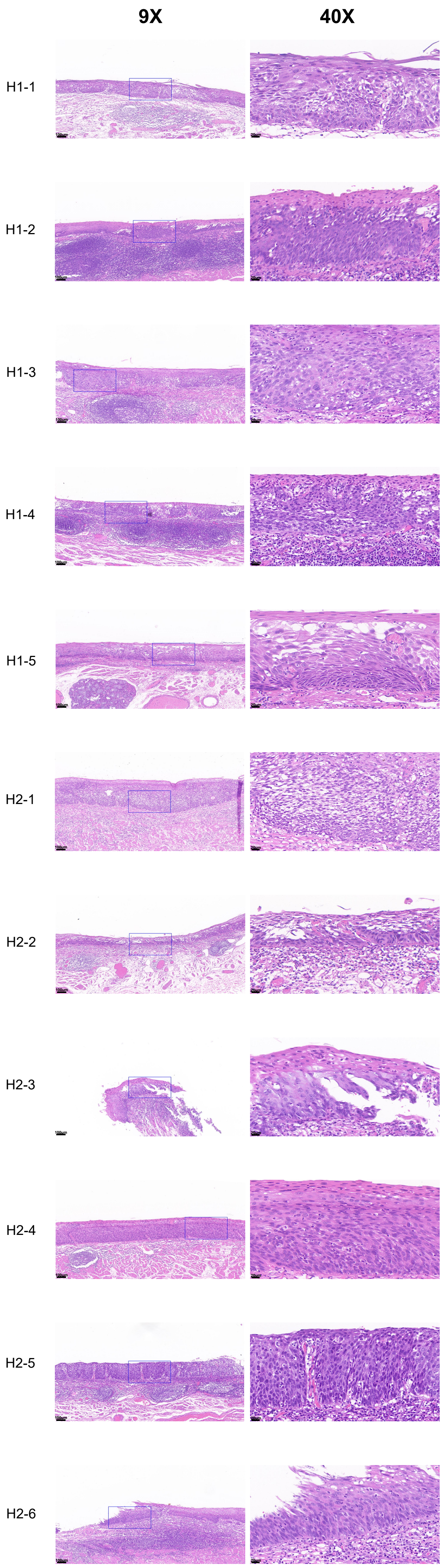

Figure S2

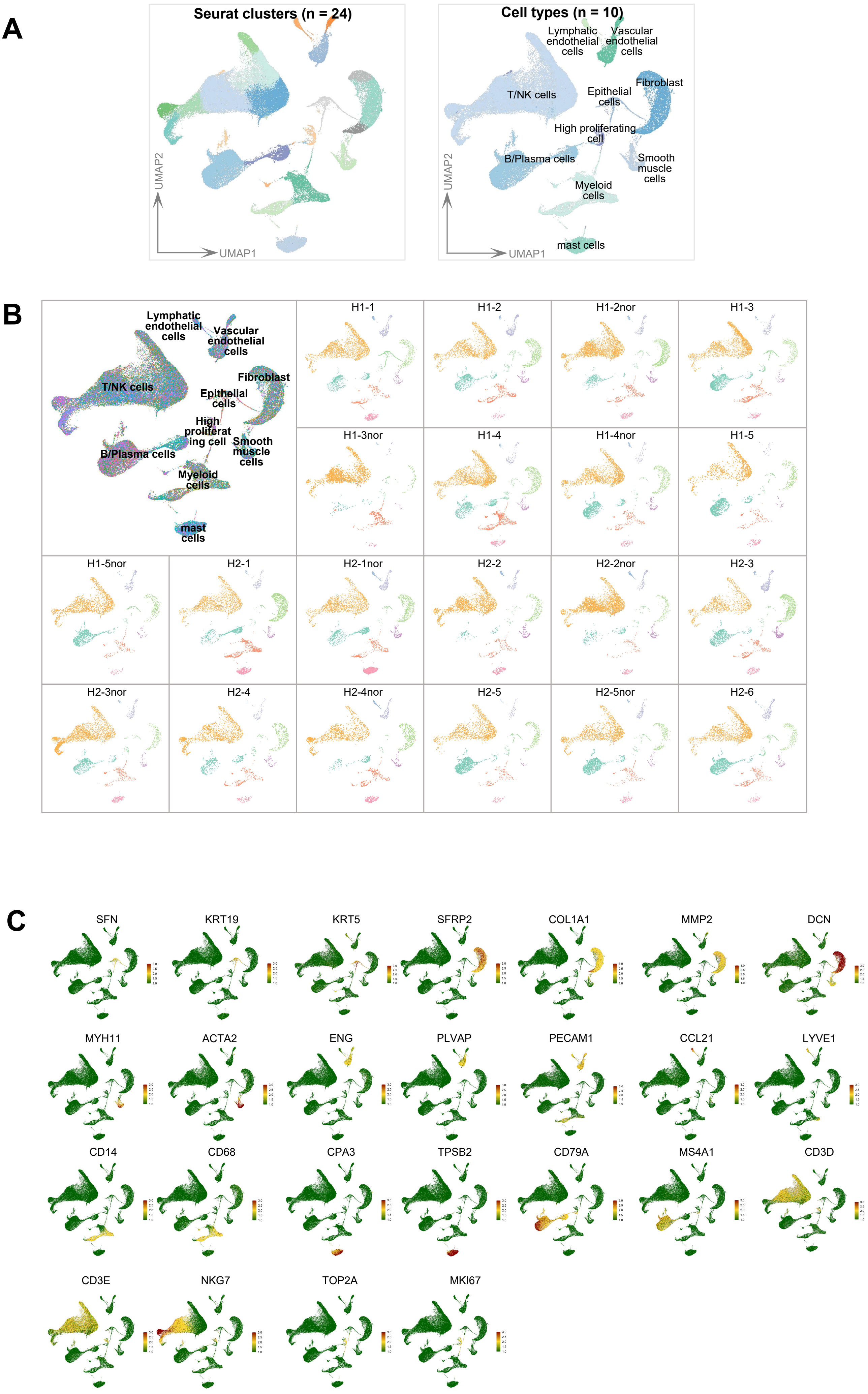
A
Seurat clusters (n = 24)
UMAP2
UMAP1
Cell types (n = 10)
Lymphatic endothelial cells
Vascular endothelial cells
T/NK cells
Epithelial cells
Fibroblast
High proliferating cell
B/Plasma cells
Smooth muscle cells
Myeloid cells
mast cells
B
Lymphatic endothelial cells
Vascular endothelial cells
T/NK cells
Epithelial cells
Fibroblast
High proliferat ing cell
Smooth muscle cells
B/Plasma cells
Myeloid cells
mast cells
H1-1
H1-2
H1-2nor
H1-3
H1-3nor
H1-4
H1-4nor
H1-5
H1-5nor
H2-1
H2-1nor
H2-2
H2-2nor
H2-3
H2-3nor
H2-4
H2-4nor
H2-5
H2-5nor
H2-6
C
SFN
KRT19
KRT5
SFRP2
COL1A1
MMP2
DCN
MYH11
ACTA2
ENG
PLVAP
PECAM1
CCL21
LYVE1
CD14
CD68
CPA3
TPSB2
CD79A
MS4A1
CD3D
CD3E
NKG7
TOP2A
MKI67

Figure S3

A

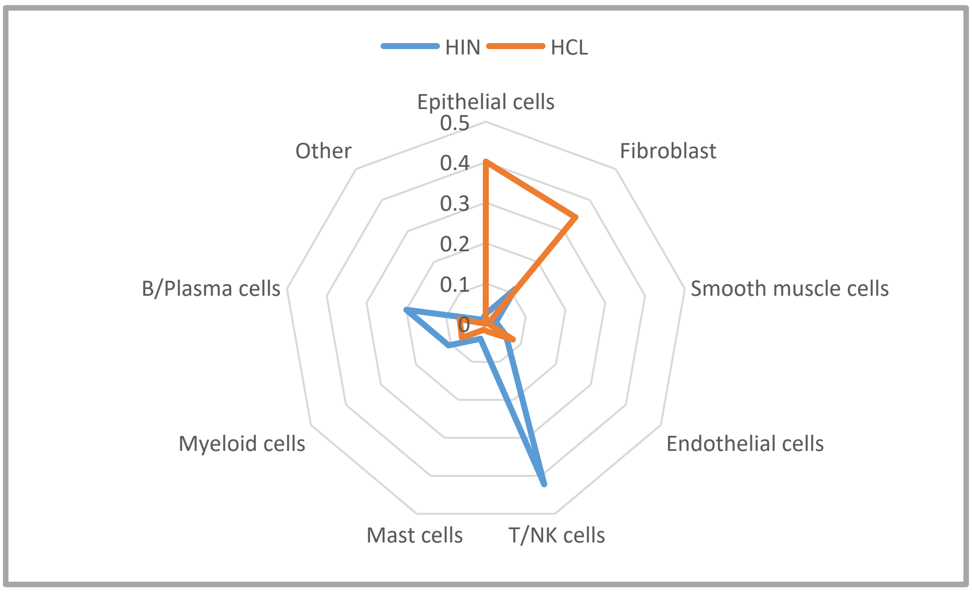

B

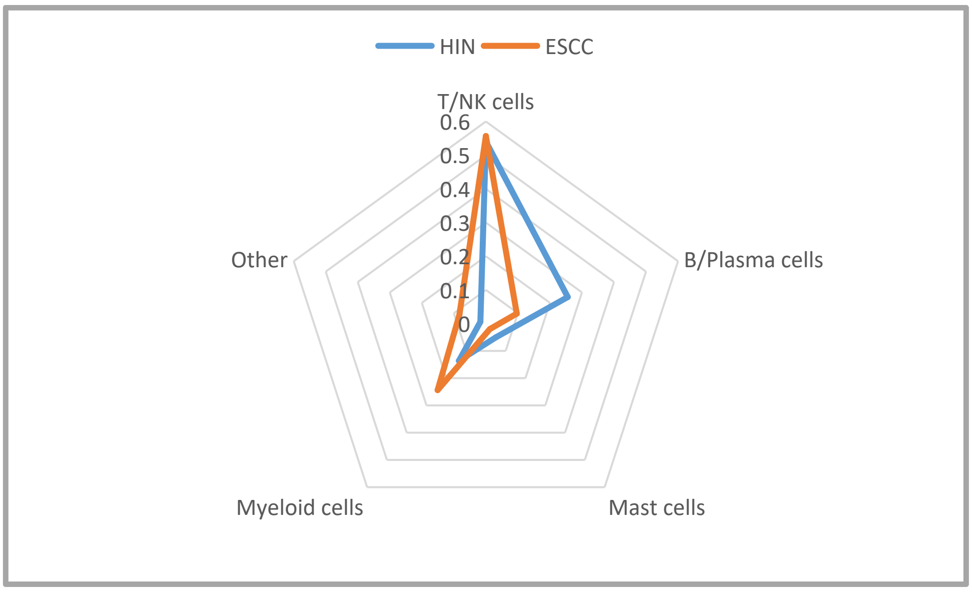

C

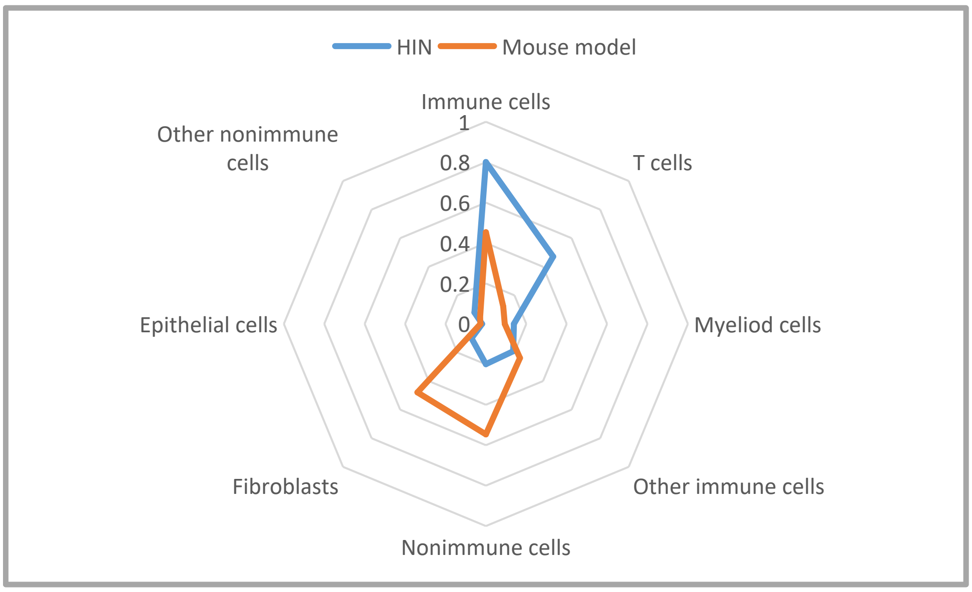

Figure S4

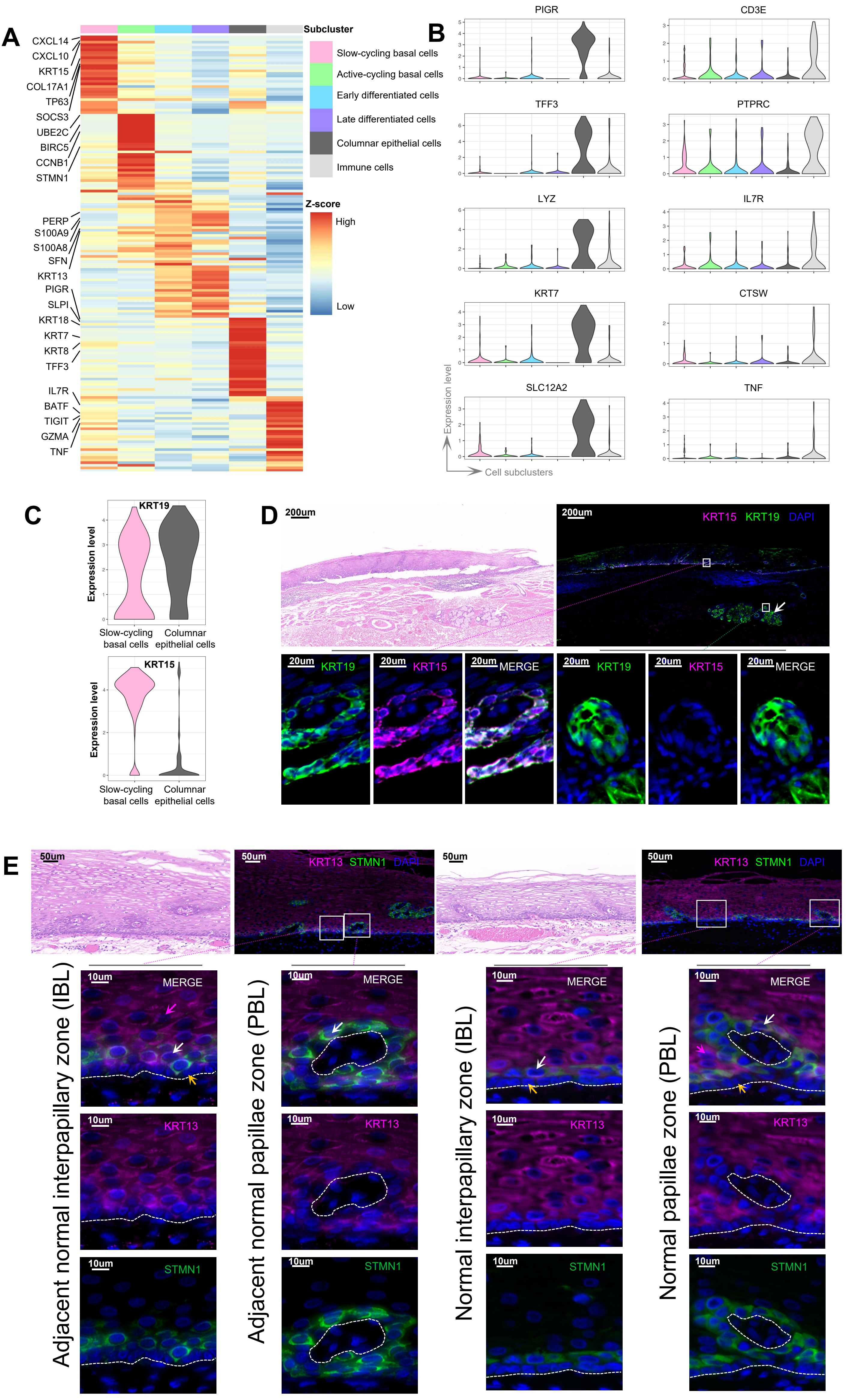
A
Subcluster
Slow-cycling basal cells
Active-cycling basal cells
Early differentiated cells
Late differentiated cells
Columnar epithelial cells
Immune cells
Z-score
High
Low
CXCL14
CXCL10
KRT15
COL17A1
TP63
SOCS3
UBE2C
BIRC5
CCNB1
STMN1
PERP
S100A9
S100A8
SFN
KRT13
PIGR
SLPI
KRT18
KRT7
KRT8
TFF3
IL7R
BATF
TIGIT
GZMA
TNF
B
PIGR
CD3E
TFF3
PTPRC
LYZ
IL7R
KRT7
CTSW
SLC12A2
TNF
Expression level
Cell subclusters
C
KRT19
KRT15
Expression level
Slow-cycling basal cells
Columnar epithelial cells
D
200um
KRT15 KRT19 DAPI
20um
KRT19
KRT15
MERGE
E
50um
KRT13 STMN1 DAPI
10um
MERGE
KRT13
STMN1
Adjacent normal interpapillary zone (IBL)
Adjacent normal papillae zone (PBL)
Normal interpapillary zone (IBL)
Normal papillae zone (PBL)

Figure S5

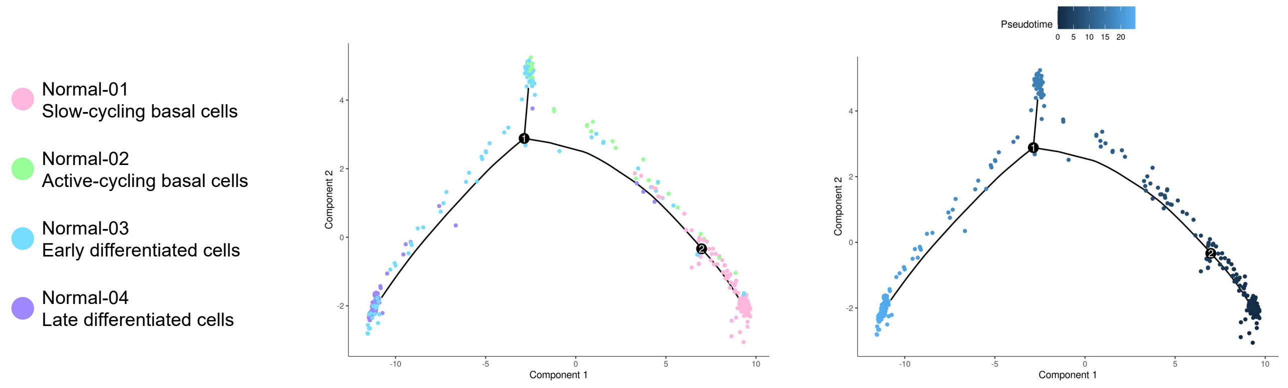

Normal-01
Slow-cycling basal cells
Normal-02
Active-cycling basal cells
Normal-03
Early differentiated cells
Normal-04
Late differentiated cells
Component 1
Component 2
Pseudotime
0 5 10 15 20
Component 1
Component 2

Figure S6

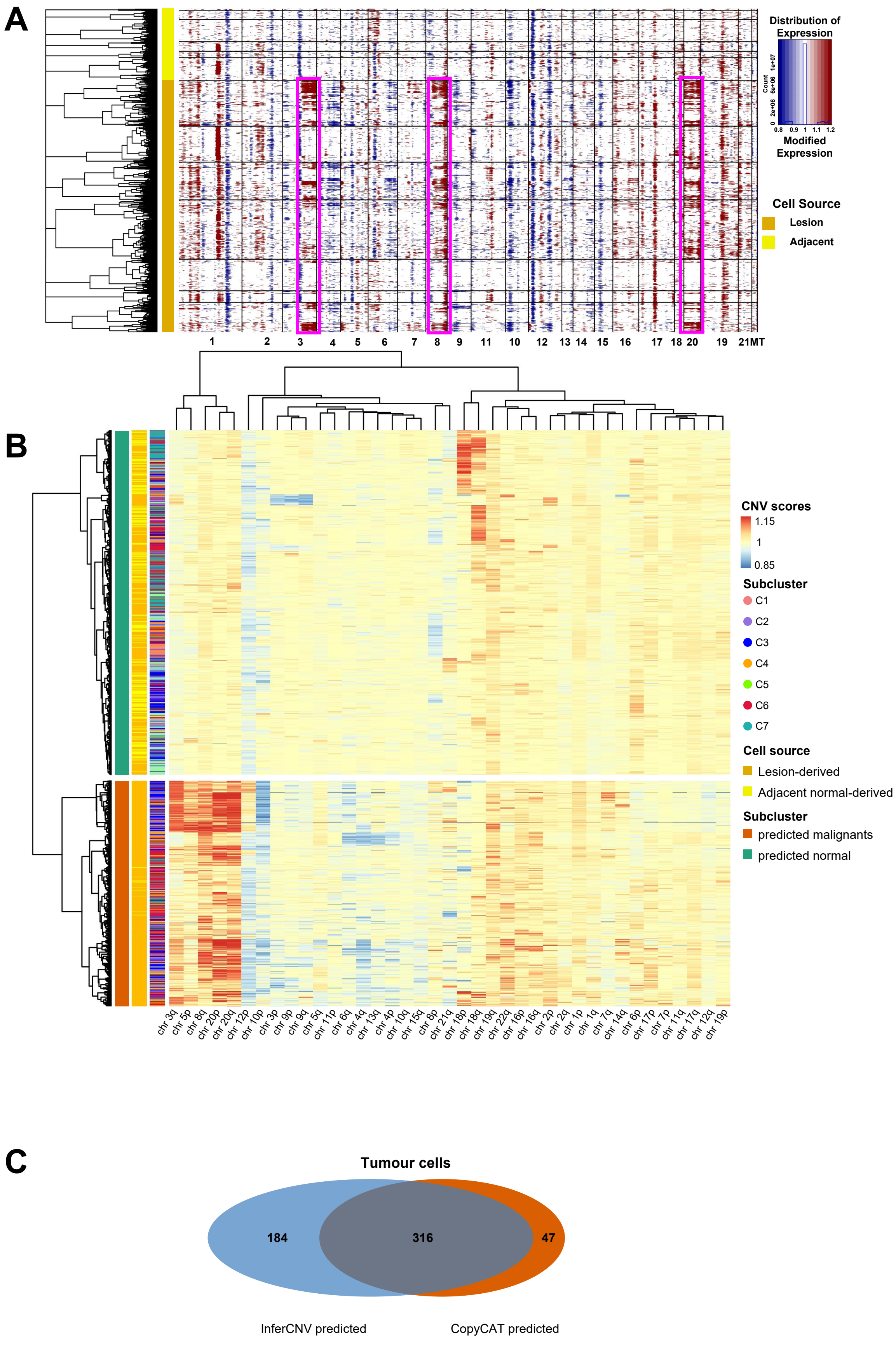
A
Distribution of Expression
Count
Modified Expression
Cell Source
Lesion
Adjacent
1 2 3 4 5 6 7 8 9 11 10 12 13 14 15 16 17 18 20 19 21 MT
B
CNV scores
1.15
1
0.85
Subcluster
C1
C2
C3
C4
C5
C6
C7
Cell source
Lesion-derived
Adjacent normal-derived
Subcluster
predicted malignants
predicted normal
chr 3q chr 5p chr 8q chr 20p chr 20q chr 12p chr 10p chr 3p chr 9p chr 9q chr 5q chr 11p chr 6q chr 4q chr 13q chr 4p chr 10q chr 15q chr 8p chr 21q chr 18p chr 18q chr 19q chr 22q chr 16p chr 16q chr 2p chr 2q chr 1p chr 1q chr 7q chr 14q chr 6p chr 17p chr 7p chr 11q chr 17q chr 12q chr 19p
C
Tumour cells
184
316
47
InferCNV predicted
CopyCAT predicted

Figure S7

A

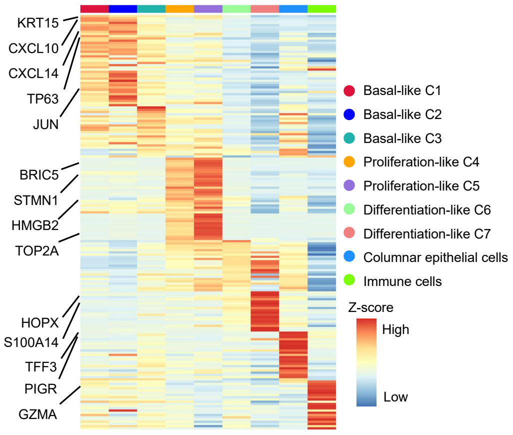

B

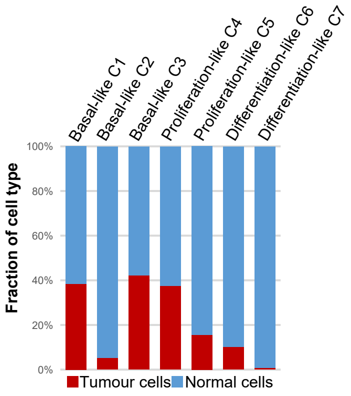

C

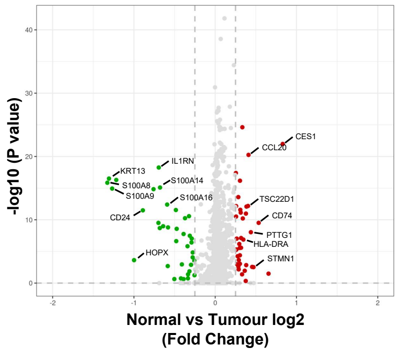

Figure S8

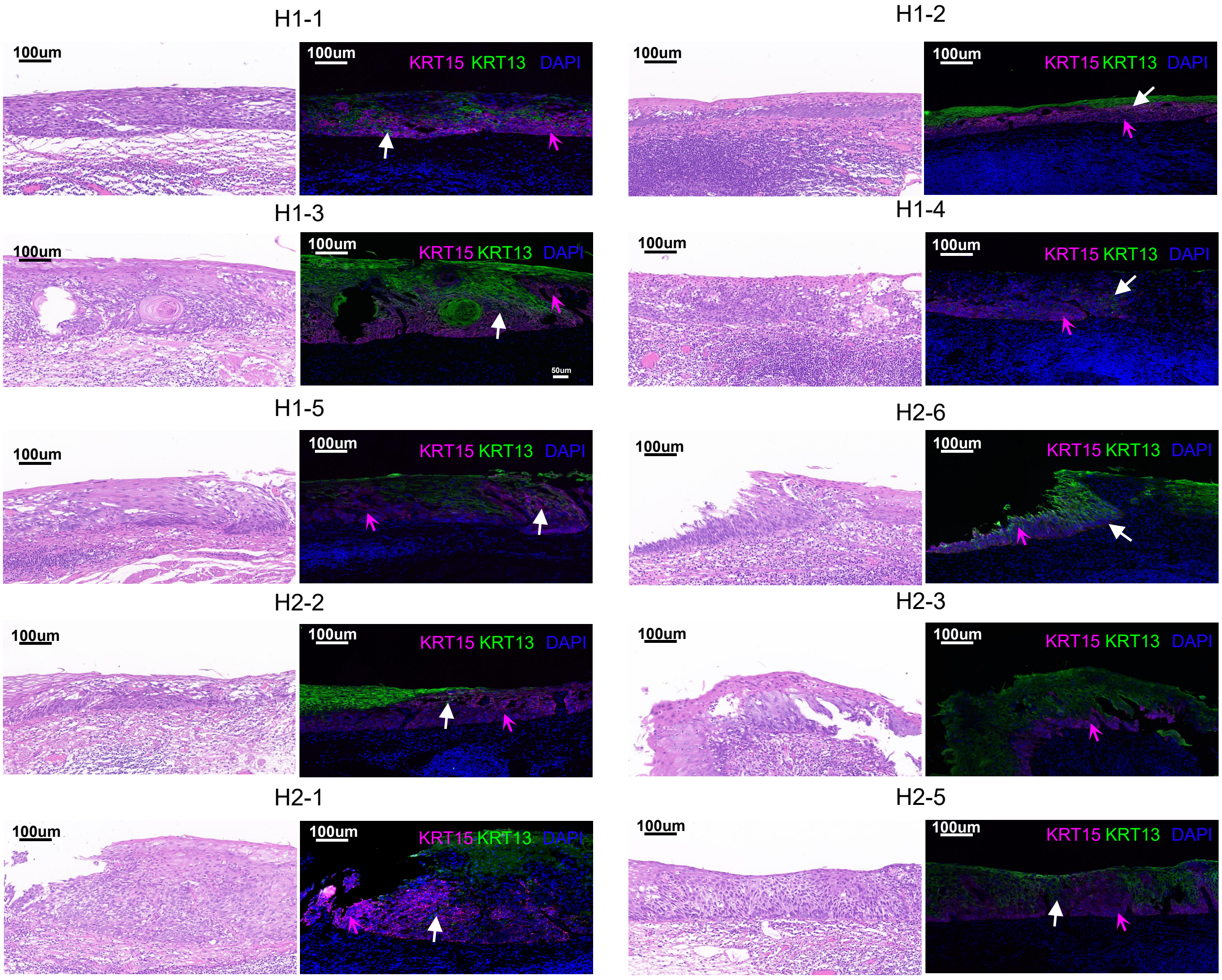
H1-1
100um
KRT15 KRT13 DAPI
H1-2
H1-3
50um
H1-4
H1-5
H2-6
H2-2
H2-3
H2-1
H2-5

Figure S9

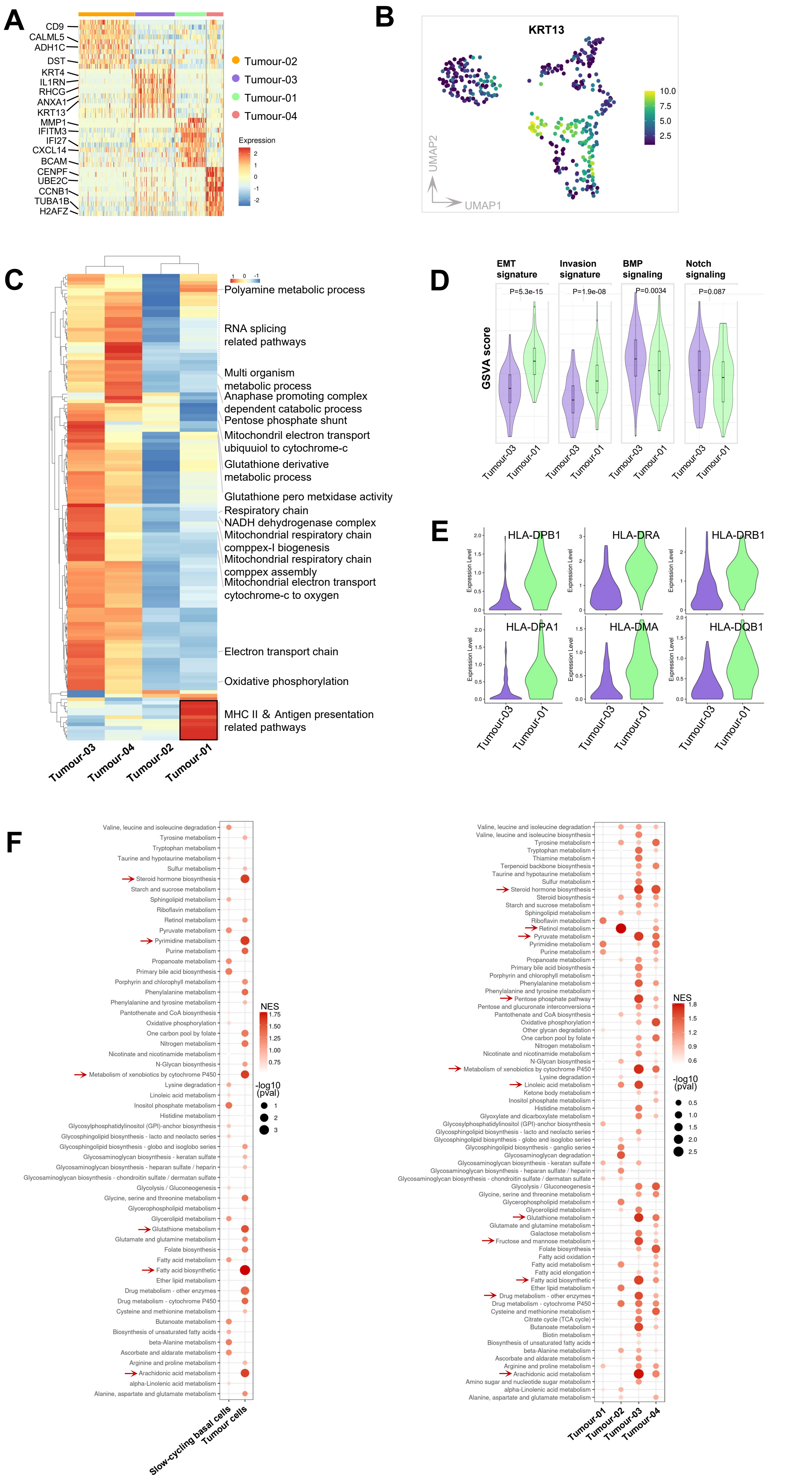
A
CD9
CALML5
ADH1C
DST
KRT4
IL1RN
RHCG
ANXA1
KRT13
MMP1
IFITM3
IFI27
CXCL14
BCAM
CENPF
UBE2C
CCNB1
TUBA1B
H2AFZ
Tumour-02
Tumour-03
Tumour-01
Tumour-04
Expression
B
KRT13
UMAP2
UMAP1
10.0
7.5
5.0
2.5
C
Polyamine metabolic process
RNA splicing related pathways
Multi organism metabolic process
Anaphase promoting complex dependent catabolic process
Pentose phosphate shunt
Mitochondril electron transport ubiquuiol to cytochrome-c
Glutathione derivative metabolic process
Glutathione pero metxidase activity
Respiratory chain
NADH dehydrogenase complex
Mitochondrial respiratory chain comppex-I biogenesis
Mitochondrial respiratory chain comppex assembly
Mitochondrial electron transport cytochrome-c to oxygen
Electron transport chain
Oxidative phosphorylation
MHC II & Antigen presentation related pathways
Tumour-03
Tumour-04
Tumour-02
Tumour-01
D
EMT signature
Invasion signature
BMP signaling
Notch signaling
GSVA score
P=5.3e-15
P=1.9e-08
P=0.0034
P=0.087
E
HLA-DPB1
HLA-DRA
HLA-DRB1
HLA-DPA1
HLA-DMA
HLA-DQB1
Expression Level
F
NES
-log10 (pval)
Slow-cycling basal cells
Tumour cells
Tumour-01
Tumour-02
Tumour-03
Tumour-04

Figure S10

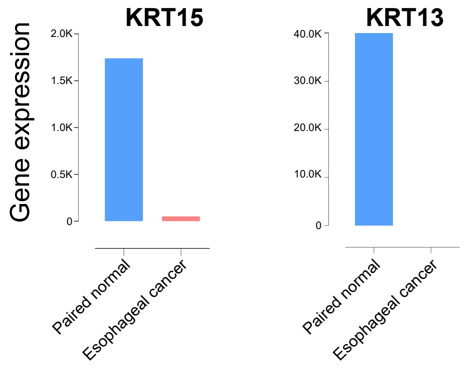
KRT15
KRT13
Gene expression
2.0K
1.5K
1.0K
0.5K
0
40.0K
30.0K
20.0K
10.0K
0
Paired normal
Esophageal cancer
Paired normal
Esophageal cancer

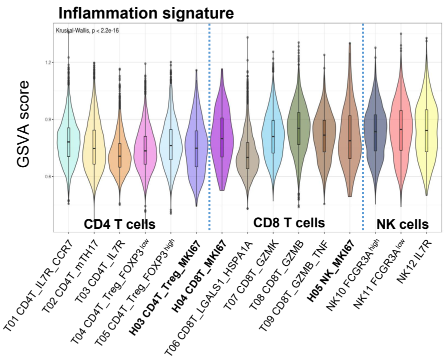
Inflammation signature
Kruskal-Wallis, p < 2.2e-16
GSVA score
1.2
0.9
0.6
CD4 T cells
CD8 T cells
NK cells
T01 CD4T_IL7R_CCR7
T02 CD4T_mTH17
T03 CD4T_IL7R
T04 CD4T_Treg_FOXP3^{low}
T05 CD4T_Treg_FOXP3^{high}
H03 CD4T_Treg_MKI67
H04 CD8T_MKI67
T06 CD8T_LGALS1_HSPA1A
T07 CD8T_GZMK
T08 CD8T_GZMB
T09 CD8T_GZMB_TNF
H05 NK_MKI67
NK10 FCGR3A^{high}
NK11 FCGR3A^{low}
NK12 IL7R

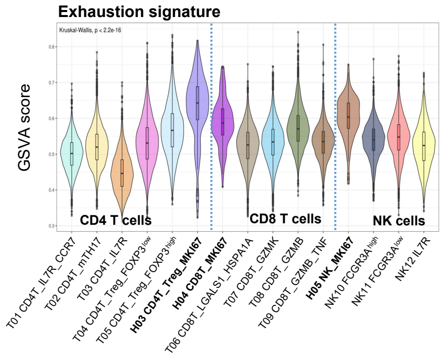
Exhaustion signature
Kruskal-Wallis, p < 2.2e-16
GSVA score
CD4 T cells
CD8 T cells
NK cells
T01 CD4T_IL7R_CCR7
T02 CD4T_mTH17
T03 CD4T_IL7R
T04 CD4T_Treg_FOXP3^{low}
T05 CD4T_Treg_FOXP3^{high}
H03 CD4T_Treg_MKI67
H04 CD8T_MKI67
T06 CD8T_LGALS1_HSPA1A
T07 CD8T_GZMK
T08 CD8T_GZMB
T09 CD8T_GZMB_TNF
H05 NK_MKI67
NK10 FCGR3A^{high}
NK11 FCGR3A^{low}
NK12 IL7R

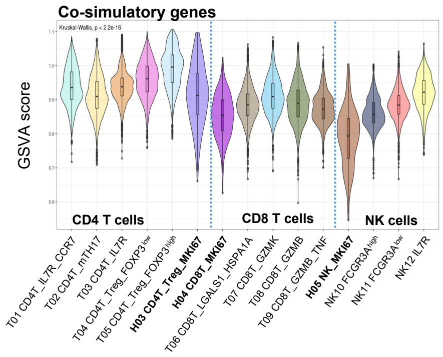
Co-simulatory genes
Kruskal-Wallis, p < 2.2e-16
GSVA score
CD4 T cells
CD8 T cells
NK cells
T01 CD4T_IL7R_CCR7
T02 CD4T_mTH17
T03 CD4T_IL7R
T04 CD4T_Treg_FOXP3^{low}
T05 CD4T_Treg_FOXP3^{high}
H03 CD4T_Treg_MKI67
H04 CD8T_MKI67
T06 CD8T_LGALS1_HSPA1A
T07 CD8T_GZMK
T08 CD8T_GZMB
T09 CD8T_GZMB_TNF
H05 NK_MKI67
NK10 FCGR3A^{high}
NK11 FCGR3A^{low}
NK12 IL7R

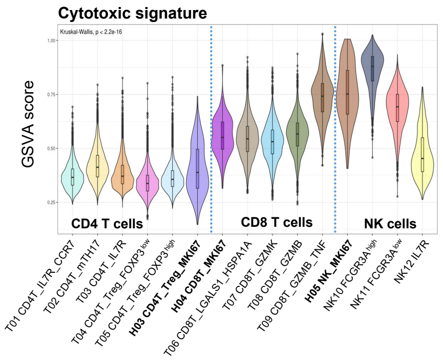
Cytotoxic signature
Kruskal-Wallis, p < 2.2e-16
GSVA score
CD4 T cells
CD8 T cells
NK cells
T01 CD4T_IL7R_CCR7
T02 CD4T_mTH17
T03 CD4T_IL7R
T04 CD4T_Treg_FOXP3^{low}
T05 CD4T_Treg_FOXP3^{high}
H03 CD4T_Treg_MKI67
H04 CD8T_MKI67
T06 CD8T_LGALS1_HSPA1A
T07 CD8T_GZMK
T08 CD8T_GZMB
T09 CD8T_GZMB_TNF
H05 NK_MKI67
NK10 FCGR3A^{high}
NK11 FCGR3A^{low}
NK12 IL7R

Figure S12

A

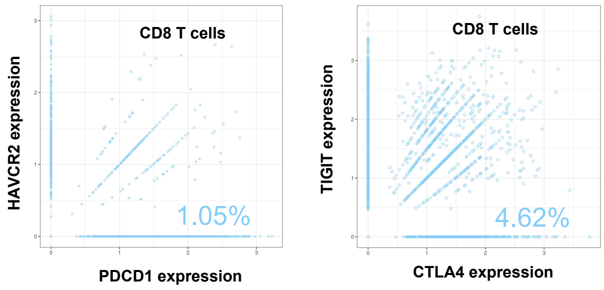

B

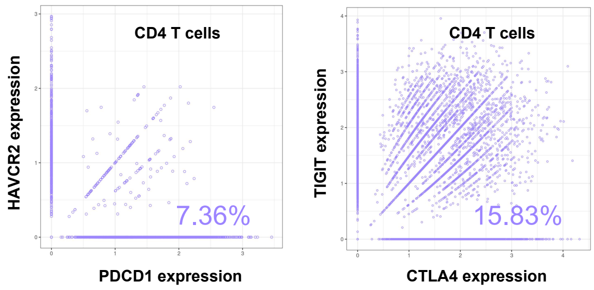

Figure S13

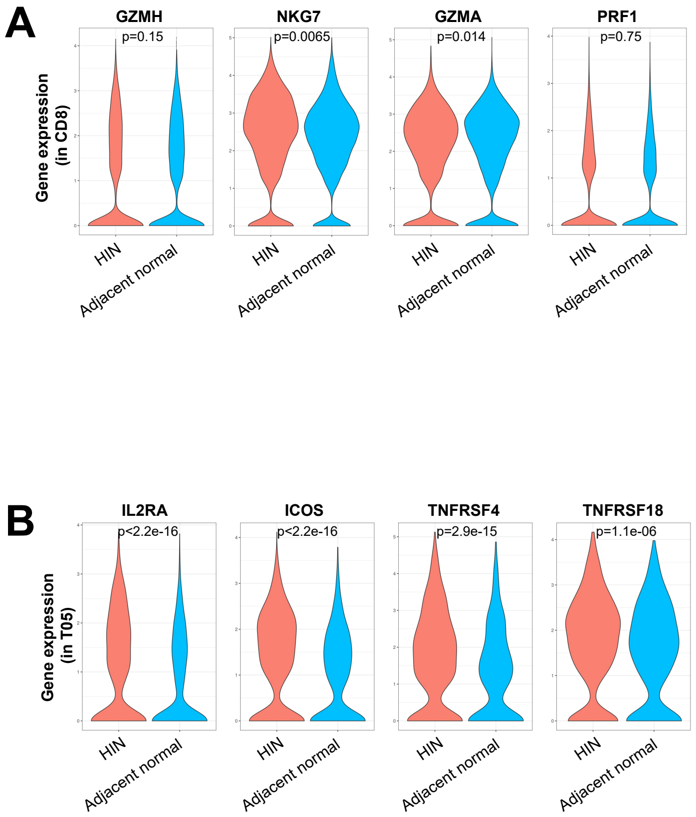
A
GZMH
p=0.15
NKG7
p=0.0065
GZMA
p=0.014
PRF1
p=0.75
Gene expression
(in CD8)
HIN
Adjacent normal
B
IL2RA
p<2.2e-16
ICOS
p<2.2e-16
TNFRSF4
p=2.9e-15
TNFRSF18
p=1.1e-06
Gene expression
(in T05)
HIN
Adjacent normal

Figure S14

A

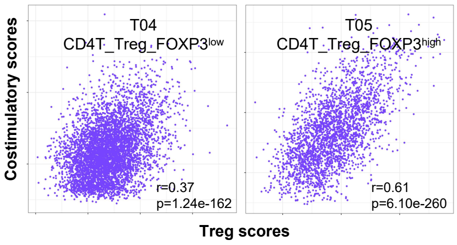

B

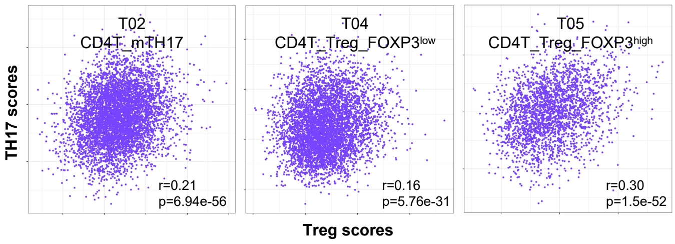

Figure S15

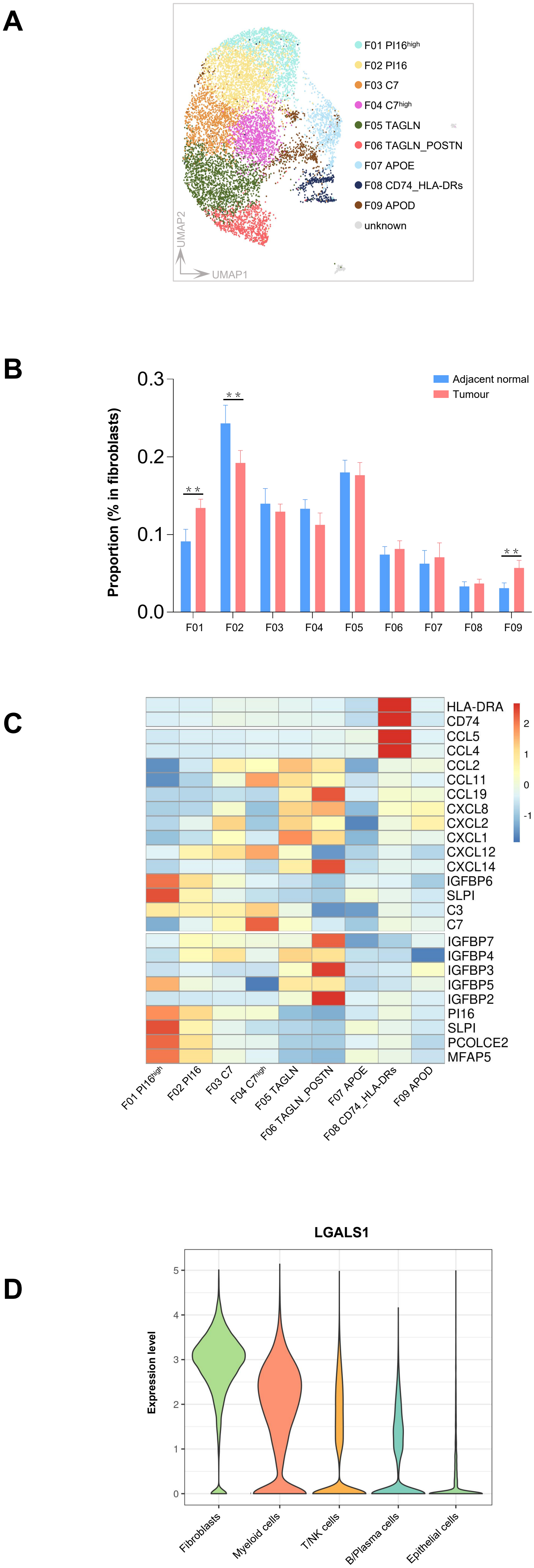
A
F01 PI16high
F02 PI16
F03 C7
F04 C7high
F05 TAGLN
F06 TAGLN_POSTN
F07 APOE
F08 CD74_HLA-DRs
F09 APOD
unknown
UMAP2
UMAP1
B
Adjacent normal
Tumour
Proportion (% in fibroblasts)
0.0
0.1
0.2
0.3
F01
F02
F03
F04
F05
F06
F07
F08
F09
C
HLA-DRA
CD74
CCL5
CCL4
CCL2
CCL11
CCL19
CXCL8
CXCL2
CXCL1
CXCL12
CXCL14
IGFBP6
SLPI
C3
C7
IGFBP7
IGFBP4
IGFBP3
IGFBP5
IGFBP2
PI16
SLPI
PCOLCE2
MFAP5
2
1
0
-1
F01 PI16high
F02 PI16
F03 C7
F04 C7high
F05 TAGLN
F06 TAGLN_POSTN
F07 APOE
F08 CD74_HLA-DRs
F09 APOD
D
LGALS1
Expression level
Fibroblasts
Myeloid cells
T/NK cells
B/Plasma cells
Epithelial cells

Figure S16

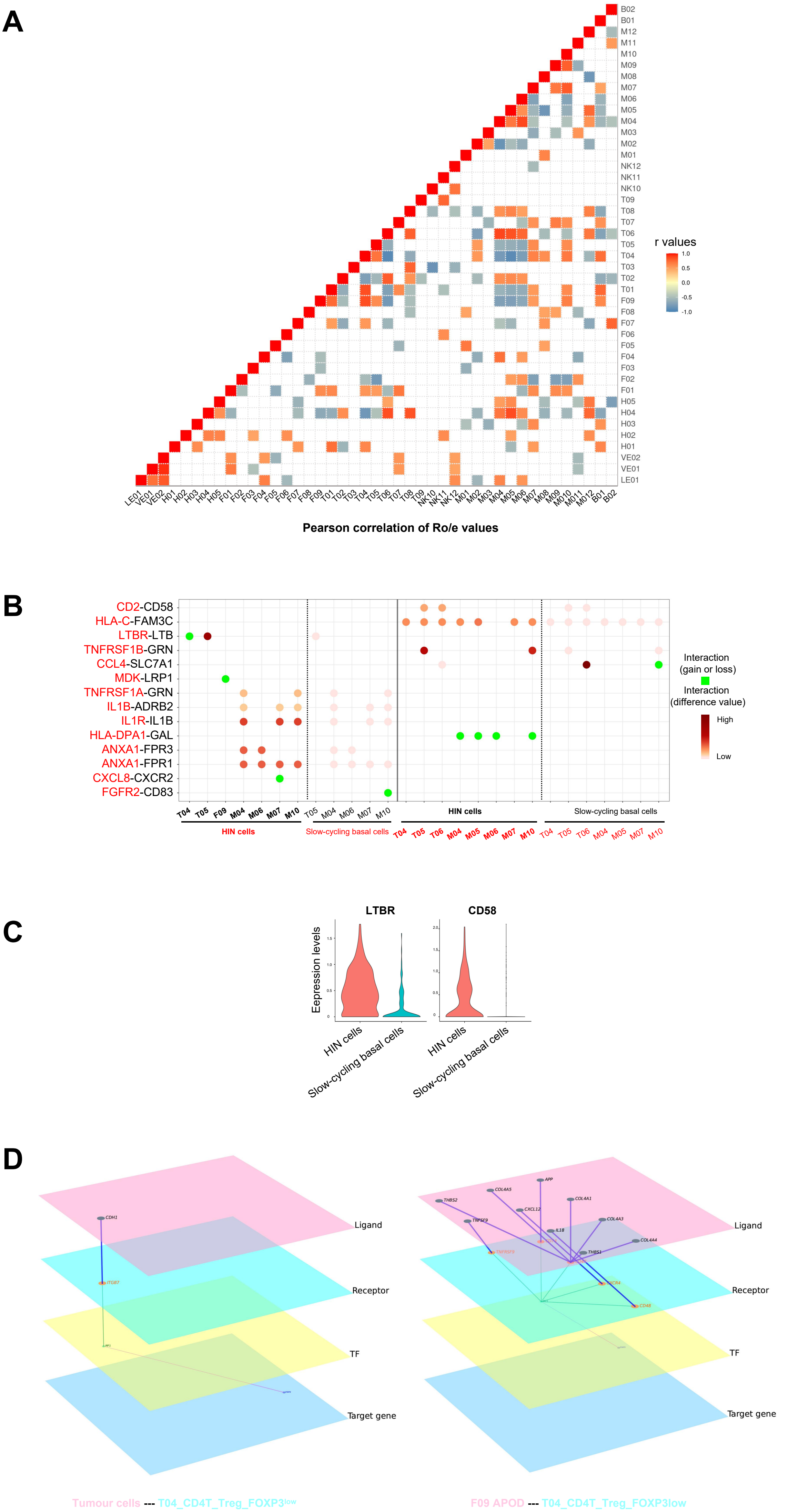
A
B02
B01
M12
M11
M10
M09
M08
M07
M06
M05
M04
M03
M02
M01
NK12
NK11
NK10
T09
T08
T07
T06
T05
T04
T03
T02
T01
F09
F08
F07
F06
F05
F04
F03
F02
F01
H05
H04
H03
H02
H01
VE02
VE01
LE01
r values
1.0
0.5
0.0
-0.5
-1.0
Pearson correlation of Ro/e values
B
CD2-CD58
HLA-C-FAM3C
LTBR-LTB
TNFRSF1B-GRN
CCL4-SLC7A1
MDK-LRP1
TNFRSF1A-GRN
IL1B-ADRB2
IL1R-IL1B
HLA-DPA1-GAL
ANXA1-FPR3
ANXA1-FPR1
CXCL8-CXCR2
FGFR2-CD83
Interaction (gain or loss)
Interaction (difference value)
High
Low
T04 T05 F09 M04 M06 M07 M10
HIN cells
T05 M04 M06 M07 M10
Slow-cycling basal cells
HIN cells
Slow-cycling basal cells
T04 T05 T06 M04 M05 M06 M07 M10
T04 T05 T06 M04 M05 M07 M10
C
LTBR
CD58
Epression levels
HIN cells
Slow-cycling basal cells
HIN cells
Slow-cycling basal cells
D
Ligand
Receptor
TF
Target gene
Ligand
Receptor
TF
Target gene
Tumour cells --- T04_CD4T_Treg_FOXP3low
F09 APOD --- T04_CD4T_Treg_FOXP3low
